# Prioritisation for future surveillance, prevention and control of 98 communicable diseases in Belgium: a 2018 multi-criteria decision analysis study

**DOI:** 10.1186/s12889-020-09566-9

**Published:** 2021-01-22

**Authors:** Sofieke Klamer, Nina Van Goethem, Working group Disease and Criteria selection, Gaetan Muyldermans, Kris Vernelen, Sarah Welby, Tommi Asikainen, Luis Campoverde, Working group Epidemiologists, Gaetan Muyldermans, Amber Litzroth, Javiera Rebolledo Gonzalez, Stéphanie Jacquinet, Martine Sabbe, Elise Mendes da Costa, André Sasse, Dominique Van Beckhoven, Nathalie Bossuyt, Tinne Lernout, Tine Grammens, Virginie Maes, Katrien Tersago, Laurence Geebelen, Daniel Thomas, Els Duysburgh, Toon Braeye, Sophie Quoilin

**Affiliations:** 1 Epidemiology and public health, Epidemiology of infectious diseases, Sciensano, Brussels, Belgium; 2 European Programme for Intervention Epidemiology Training (EPIET), European Centre for Disease Prevention and Control (ECDC), Stockholm, Sweden; 3 Communicable Disease Surveillance Centre, Public Health Wales, Cardiff, Wales; 4 Epidemiology and public health, Healthcare-associated infections, Sciensano, Brussels, Belgium

**Keywords:** Infectious diseases, Prioritisation, MCDA, Allocation of resources, Ranking, Burden of disease, Health domains, Expert perspectives, Surveillance priorities, Public health action

## Abstract

**Background:**

National public health agencies are required to prioritise infectious diseases for prevention and control. We applied the prioritisation method recommended by the European Centre for Disease Prevention and Control to rank infectious diseases, according to their relative importance for surveillance and public health, to inform future public health action in Belgium.

**Methods:**

We applied the multi-criteria-decision-analysis approach. A working group of epidemiologists and statisticians from Belgium (*n* = 6) designed a balanced set of prioritisation criteria. A panel of Belgian experts (*n* = 80) allocated in an online survey each criteria a weight, according to perceived relative importance. Next, experts (*n* = 37) scored each disease against each criteria in an online survey, guided by disease-specific factsheets referring the period 2010–2016 in Belgium. The weighted sum of the criteria’s scores composed the final weighted score per disease, on which the ranking was based. Sensitivity analyses quantified the impact of eight alternative analysis scenarios on the top-20 ranked diseases. We identified criteria and diseases associated with data-gaps as those with the highest number of blank answers in the scoring survey. Principle components of the final weighted score were identified.

**Results:**

Working groups selected 98 diseases and 18 criteria, structured in five criteria groups. The diseases ranked highest were (in order) pertussis, human immunodeficiency virus infection, hepatitis C and hepatitis B. Among the five criteria groups, overall the highest weights were assigned to ‘impact on the patient’, followed by ‘impact on public health’, while different perceptions were identified between clinicians, microbiologists and epidemiologists. Among the 18 individual criteria, ‘spreading potential’ and ‘events requiring public health action’ were assigned the highest weights. Principle components clustered with thematic disease groups. Notable data gaps were found among hospital-related diseases.

**Conclusions:**

We ranked infectious diseases using a standardised reproducible approach. The diseases ranked highest are included in current public health programs, but additional reflection for example about needs among risk groups is recommended. Cross-reference of the obtained ranking with current programs is needed to verify whether resources and activities map priority areas. We recommend to implement this method in a recurrent evaluation cycle of national public health priorities.

## Background

Funding for health protection services is finite. Decisions as how best to direct resources to maximise public health impact are therefore essential. However, national public health agencies struggle to prioritise infectious diseases for surveillance, prevention and control. The allocation of human and material resources is often based on residuals of historic situations, short term political agendas or individual preferences [1]. In Belgium a clear and reproducible strategy for this prioritisation is lacking. A standardised, transparent and reproducible approach may guide and justify allocation strategies within public health programs and might result in a more cost-effective and impactful public health service [1, 2].

Policy and decision makers can use single indicators to make informed decisions about communicable diseases, like the reported incidence, the estimated population incidence or mortality rates. In addition, composite indicators can be used, for example the standardised burden indicators expressed in disability adjusted life years (DALY), which represent the sum of morbidity and mortality associated to specific diseases. Burden indicators are excellent quantifiers of the impact at the level of the individual patient and its extrapolation to the population, but do not include important aspects related to communicable diseases, such as the specific impact of communicability and the need of preparedness and coordinated response. Multi-criteria decision analysis (MCDA) takes into account a flexible set of indicators, that is tailored towards the agreed objective of the analysis. MCDA has previously been applied to a communicable disease priority setting in a number of countries of the European Union (EU) [3–6]. The European Centre of Disease Prevention and Control (ECDC) has recommended the use of MCDA or similar methods in recurrent evaluation cycles of national priority setting for management of infectious diseases [7].

In Belgium, MCDA was previously applied to rank zoonoses, based on their impact on humans, animals and the economy [8–10]. A more general approach for a broader range of communicable diseases was not yet described in Belgium. We aimed to rank communicable diseases according to their relative importance for surveillance and public health, in order to inform future planning and allocation of resources for public health action in Belgium. We applied the ECDC-recommended prioritisation methodology [11] to the Belgium context of 2018. Analysis of the probability of introduction, spread and establishment of emerging diseases [12] was outside the scope of our objective. A secondary objective was to identify the components that compose the final weighted scores per disease, in order to understand which variables contribute the most to disease priority.

Here, we described a transparent prioritisation process according to standardised and well-documented methods, which may initiate an inter-disciplinary reflection about priority setting strategies for communicable disease control programs in Belgium. We intended to engage public health clinicians, public health workers, decision- and policy makers of different administrative levels and scientific institutes across Belgium to set evidence-based priorities in the domain of public health in Belgium.

## Methods

**Fig. 1:**
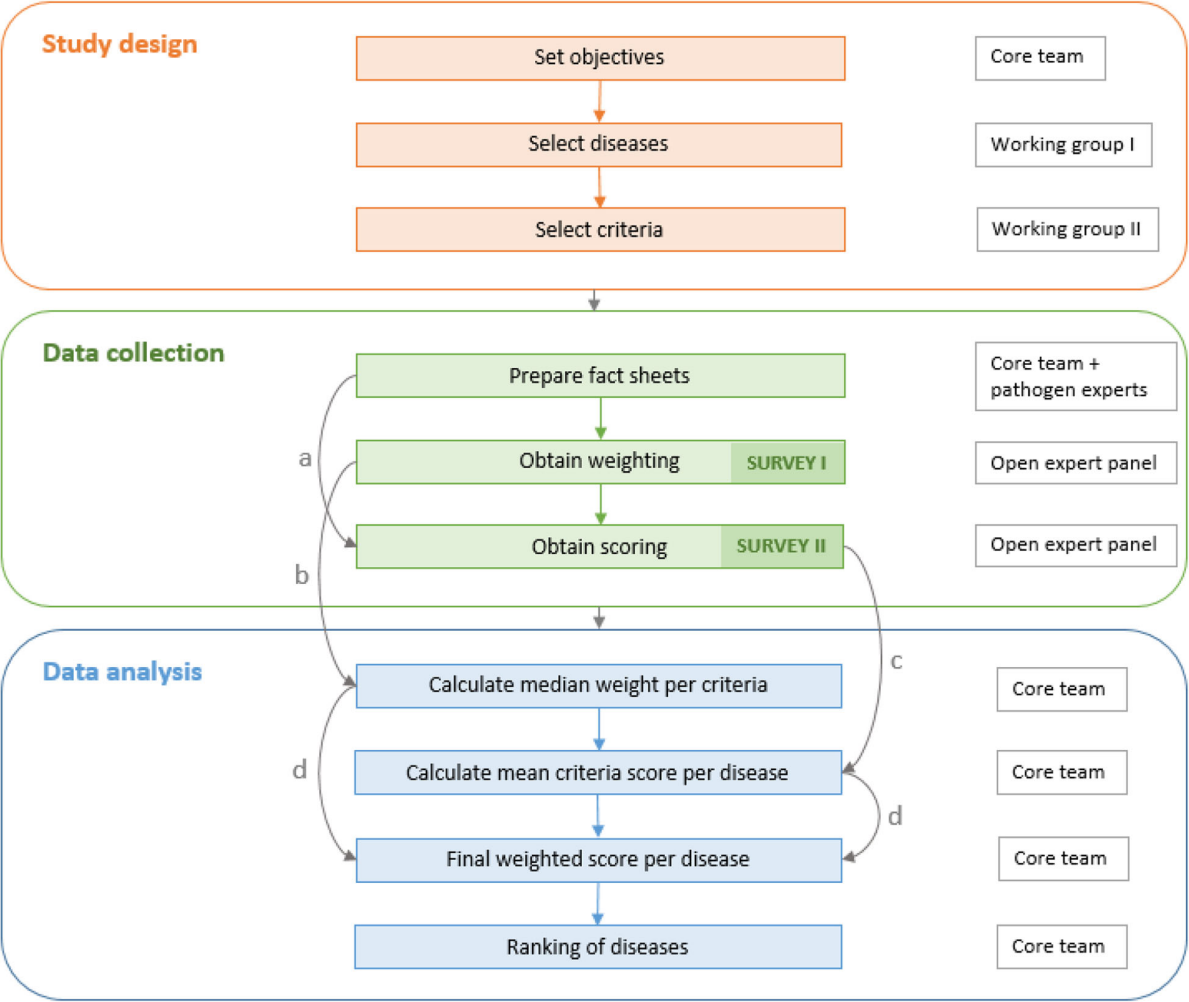
Overview of the study methodology. The figure shows the study design (orange), data collection (green) and data analysis (blue) phases and the persons/teams involved in each phase (white boxes). The position of the two surveys, the weighting survey and the scoring survey, are indicated in dark green boxes. Arrows indicate dependencies. **a** Fact sheets (evidence-based data) was provided to accompany the scoring survey and to set default answers within this scoring survey (survey II). **b** Median weights per criteria were calculated using expert responses (survey I). **c** Mean criteria scores per disease were calculated based on the expert responses (survey II). **d** The weighted sum of the criteria scores composed the final weighted score per disease, on which the ranking was based

We used the MCDA approach in the Belgian context of 2018 and sequentially applied the following steps: selection of infectious diseases in a working group, selection of prioritisation criteria in a second working group, weighting of the criteria by an open panel of experts in a first online survey (Additional file 2: Survey-I), scoring of the diseases for each criteria in a second online survey (Additional file 3: Survey-II) and, finally, calculating the final weighted score on which the ranking of diseases was based (Fig. 1).

### Selection of diseases

Diseases were selected within a working group of seven members, consisting of epidemiologists and microbiologists from the Belgian institute of health (Sciensano). We choose to describe the selected diseases as diseases provoked by one single pathogen, instead of classification by clinical syndromes or by groups of pathogens with certain characteristics (e.g. multidrug resistance). When the pathogen provokes a spectrum of diseases, we choose to focus on the more severe manifestations of disease, for example the congenital diseases and bloodstream infections. The first step in the disease selection process applied four objective criteria. Diseases were selected when fulfilling at least one of the following criteria: (1) notifiable by law in at least one of the three Belgium regions; (2) a national reference centre (NRC) established by law in Belgium or a voluntary reference laboratory existing in Belgium; (3) reportable within the EU or reportable to the World Health Organisation (WHO) or World Organisation for Animal Health (OIE); or (4) being identified as highest priority (a score of 75% or higher) in the latest German prioritisation study [5]. Next, an extended list of yet unselected diseases was prepared, inspired by diseases included in other prioritisation studies [5] and extended by disease proposals from the working group members (*n* = 7). We considered diseases that are not selectively affecting immunocompromised persons. Furthermore, we excluded diseases for which we agreed that clinical presentation was very mild in otherwise healthy persons (for example ‘head lice’). In addition, diseases that were not detected or suspected to occur within our country were excluded from our analysis (for example ‘rift valley fever’), because the risk of introduction was outside the study objective. However, diseases without occurrence but with an impact on current disease management programs (for example polio and rabies) or a high media impact (for example ebola) were included. A scoring of all diseases was done by the working group members individually (1 = not to include; 2 = maybe include; 3 = include). Diseases were included that obtained on average two or more of the three available points. For the sake of clarity, the list of diseases was presented in the survey in five thematic disease groups: vaccine-preventable diseases, endemic diseases, imported/rare diseases, diseases with limited surveillance or congenital risks, hospital-related diseases. Diseases that fitted in multiple disease groups, were classified in just one thematic disease group.

### Selection of prioritisation criteria

Next, prioritisation criteria were selected: a balanced set of criteria structured in criteria groups, was composed by a working group of epidemiologists, microbiologists and statisticians from Sciensano (six members, partly overlapping with the disease selection working group). The main criteria groups were inspired by the ECDC prioritisation tool [11] and the German prioritisation study [5]. However, we selected a higher number of quantitative criteria, and we excluded criteria that exclusively focused on research. All working group members could propose prioritisation criteria, which subsequently were organised in criteria groups. Finally, the working group selected in consensus discussion the most relevant criteria groups and criteria within each group (Additional file 1: Table S1), avoiding overlap between criteria. Detailed criteria definitions were formulated (Additional file 1) and answer categories with corresponding quantifying scales were defined (linear or logarithmic; Additional file 1: Table S2).

### Data collection: criteria weights and disease scores

Two anonymous online surveys were performed to collect expert opinions (perceived weights of the criteria and criteria scores per disease). No medical or personal data was processed. Participants were informed about the objective and design of the study and their rights before participation to the survey. A first survey was conducted among a panel of key informants to obtain criteria weights according to the perceived relative importance. The participants were asked to assign a weight between 1 and 10 to each individual criteria within its criteria group. Next, the participants were asked to rate the relative importance of the main criteria groups, by assigning the criteria groups a weight between 1 and 10. Internal validity within this survey was tested by a seed question (Additional file 2: section D): participants were asked to rank three sub-criteria, and this ranking was compared to the ranking that resulted from their answers in the previous questions for the same sub-criteria. The impact of this validity test was analysed in analysis scenario F (see below).

A second survey was distributed after closure of the first survey. This second survey asked the experts to score each disease against the individual criteria, guided by surveillance and background data presented in fact sheets. These disease-specific fact sheets were prepared in collaboration with the relevant pathogen experts within Sciensano, together constituting the epidemiologists working group. For each selected disease, data was collected concerning the reference period 2010–2016 and concerning the Belgian context, in order to provide evidence to the panel of experts during the scoring of the diseases. Data was collected using text book information, recent surveillance reports and existing scientific literature. In addition, the collected evidence was used to set default answers for some of the criteria within the scoring survey (Additional file 1: Table S1). The values for the default answers were set by at least two epidemiologists independently, and conflicting values were discussed until consensus was reached. For convenience purposes, the diseases were presented in the five thematic disease groups within the survey and respondents had the possibility to skip questions (criteria) for complete disease groups.

The two surveys were developed in LimeSurvey (Version 2.71.1) and were pre-tested for acceptability and intelligibility by three researchers not directly involved in the development phase. Invitation letters, including the link to the online survey, were distributed by email to members of the Belgian Society of Infection Specialists and Clinical Microbiologists (BVIKM), physicians of the regional infectious disease control teams, physicians participating to the paediatric infectious disease surveillance network, microbiologists participating in sentinel surveillance, other clinical microbiology laboratories, the audience of the infectious disease monthly news bulletin, public health experts of the national antimicrobial resistance working group, public health experts within the ministry of health and the scientific public health institute (Sciensano). The same target group was considered for both surveys.

### Data analysis: criteria weights

Median hierarchical weights for each criterion were calculated in three steps, based on the raw responses obtained in the first survey that aimed to obtain weights. In the first step, the raw survey results were rescaled at the level of the individual response by dividing by the mean within each question (unit normalisation), resulting in a centring around one of the individual respondents weights. Secondly, the individual scores were calculated per criterion at the individual expert level by multiplying the rescaled weight of the individual criteria with the rescaled weight of the criteria group and, subsequently, with the inverse of the number of criteria within the group. In the third step, the hierarchical weights per criterion were aggregated over the expert panel by taking the median of the scores per criterion. Alternatively, non-hierarchical weights per criterion were calculated by direct multiplication of the rescaled weight of the individual criteria with the rescaled weight of the criteria group, without taking into account the number of criteria within a group (see the second step above), and these weights were used in an alternative analysis scenario (scenario G).

### Data analysis: disease scores and priority groups

Next, we took the raw individual responses of the second survey, that aimed to obtain disease and criteria scores, and aggregated these over the expert panel by taking the average, thus obtaining the mean scores per criteria and per disease. To obtain the final weighted score, the mean criteria scores per disease were multiplied with the criteria weights, and the weighted sum of the criteria scores composed the final weighted score per disease, on which the ranking was based. Standard errors of the mean were calculated for the final weighted score per disease, summarising the variation among the respondents of the scoring survey. Based on the final weighted score and the consequent ranking, the diseases were classified in priority groups: to define the number of priority groups and the accompanying threshold values of the final weighted score, we used classification and regression tree (CART) modelling as implemented in the RPART package (R software), which aims to minimize the within-group variance [13]. The final weighted score was used as response variable and the ranking as explanatory variable in the CART regression, while using standard settings and a weighting equal to the ranking.

### Analysis scenarios and additional data analyses

**Fig. 2:**
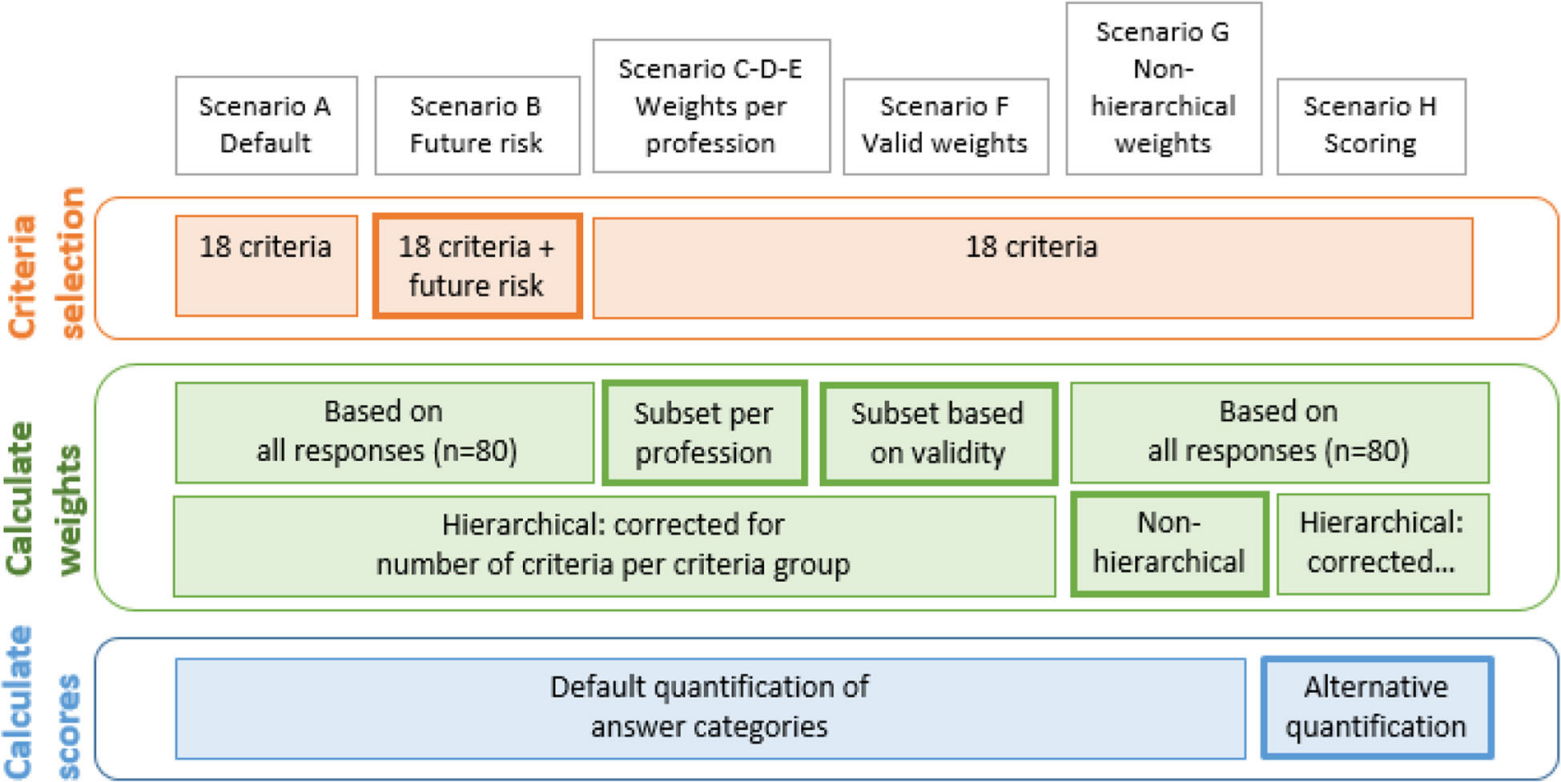
Overview of alternative analysis scenarios. Scenarios are based on alternative criteria selection (scenario B) or alternative weightings, including weighting according to professional background (scenario C, D and E), weighting only including consistent responses based on the validation question in survey I (scenario F) and non-hierarchical weighting in which the weights were not compensated for the number of criteria per group (scenario G). The final analysis scenario used alternative scales to quantify the answer categories from the scoring survey (scenario H). Boxes with bold borders represent deviations from the default analysis approach

Eight alternative analysis scenarios were considered in a sensitivity analysis, based on an alternative set of criteria, alternative weights or an alternative quantification of answer categories in the scoring survey (Fig. 2). An alternative set of criteria was used in scenario B and included ‘future risk’ in addition to the criteria used in the default scenario. The alternative weights for the criteria concerned a subset of expert responses according to the experts’ profession (scenario C-E), a subset of responses concerning only participants who passed the validation question (scenario F), or the use of non-hierarchical weights (scenario G). Finally, the last alternative scenario took into account an alternative quantification (scaling) of the answer categories in the scoring survey (Additional file 1: Table S2, scenario H). The resulting ranking were compared by a bump chart (Tableau software).

A principal component analysis (PCA) identified the relative contribution of each criteria group to the final weighted score per disease. Variables were centred around the mean and scaled to unit variances, prior to the PCA calculations. Clustering of the thematic disease groups was visualised in the PCA analysis (package ‘ggbiplot’ and ‘factoextra’). Further correlations between the individual criteria were analysed by the construction of a correlogram, based on the individual weighed criteria score per disease (package ‘corrplot’). The relative priority of the thematic disease groups was visualised in a treemap based on the final weighted score (package ‘treemap’). To visualise health domain perspectives, the weighted scores per disease were plotted for the dimensions ‘patient impact’, ‘society impact’ and ‘Public Health (PH) impact’, while ‘PH impact’ in this case represented the sum of the two criteria from ‘impact on PH’ together and the seven criteria of ‘surveillance needs’ (package ‘scatterplot3d’). Possible data gaps were identified as the thematic disease groups or criteria with the highest number of blank responses in the scoring survey or the highest standard errors in the final weighted score. Data analysis was performed using R software version 3.4.1 and Tableau 2019 2.3.

## Results

### Pathogen and criteria selection

The disease selection working group created a short-list of 129 diseases, of which 61 were selected based on objective criteria and an additional 37 were selected in the voting procedure, resulting in 98 diseases for the ranking exercise. For convenience and visualisation purposes, the 98 diseases were classified in five thematic disease groups: vaccine-preventable diseases (*n* = 15), endemic diseases (*n* = 38), imported/rare diseases (*n* = 23), diseases with limited surveillance or congenital risks (*n* = 12), hospital-related diseases (*n* = 10). The criteria selection working group short-listed 30 criteria and subsequently selected 18 prioritisation criteria to be used in the MCDA analysis, which were structured in five main criteria groups: ‘incidence and trend’, ‘impact on patient’, ‘impact on society’, ‘impact on public health’ and ‘surveillance needs’ (Table 1). The criterion ‘future risk’ was not selected for the main analysis due to the lack of objective data, but was included in an alternative analysis scenario (scenario B).

**Table 1:** Prioritisation criteria and criteria groups, the corresponding perspective and question type used in the survey

Criteria group	Criteria	Perspective	Question type
Incidence and trend	Incidence	Population	Default answers
	Trend	Population	Default answers
Impact on the patient	Case fatality ratio	Individual	Default answers
	Severity	Individual	Default answers
	Chronicity	Individual	Default answers
Impact on society	Absenteeism	Population	Open expert opinion
	Health care utilization	Population	Open expert opinion
	Excess costs	Population	Open expert opinion
	Public attention	Population	Open expert opinion
Impact on public health	Spreading potential	Pathogen	Default answers
	Events requiring PH action	Pathogen	Open expert opinion
Surveillance needs	International surveillance obligations	Pathogen	Review
	WHO objective for eradication	Pathogen	Review
	Vaccine included in NVP	Pathogen	Review
	Risk for vaccine-triggered strain replacement	Pathogen	Review
	Existing antibiotic multidrug resistance	Pathogen	Review
	NRC/Reflab essential for diagnosis	Pathogen	Review
	Congenital risks	Pathogen	Review

### Criteria weights

**Fig. 3:**
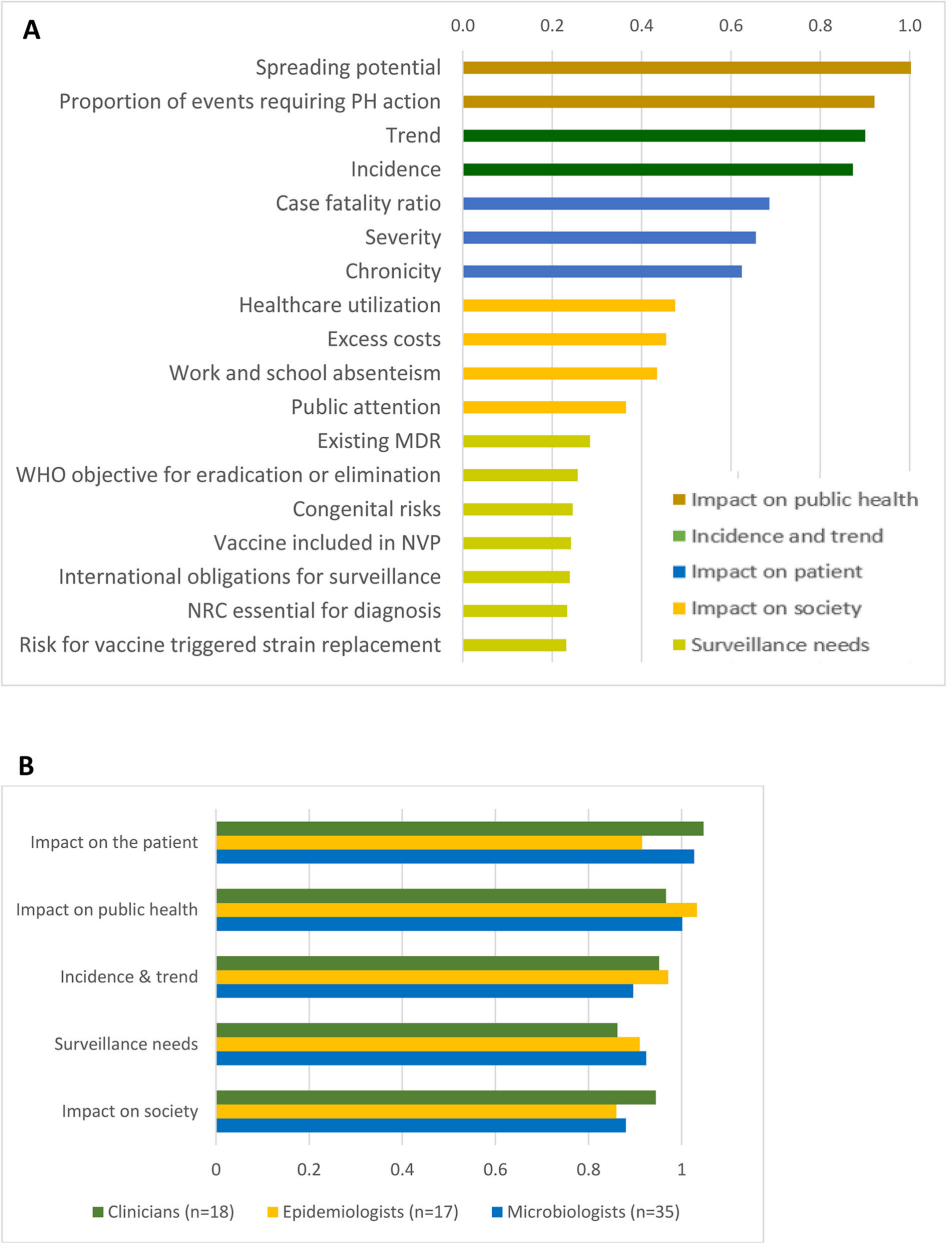
Weights of the 18 individual criteria and the five criteria groups per profession. **a** Individual criteria weights, presented relatively to the criteria that was attributed the highest weight (‘spreading potential’). Note that individual criteria weights were corrected for the number of criteria within the group, therefore criteria weights from different groups should be compared with caution. **b** Weights of the criteria groups by professional background of the responding experts, presented relatively to the criteria group that was attributed the highest weight while considering all expert responses (‘impact on the patient’)

We received 80 responses in the first survey, from 35 microbiologists, 18 clinicians, 17 public health professionals and 10 others (of whom the majority infection control nurses). Response rates could not be calculated as no personal invitations were send. Among the 18 individual criteria, the highest hierarchical weights were assigned to ‘spreading potential’ and ‘events requiring public health action’ (Fig. 3a). Among the five criteria groups, the highest weights were assigned to ‘impact on the patient’, followed by ‘impact on public health’ (Fig. 3b). Differences in perceived weight were identified between clinicians, microbiologists and epidemiologists, with for example clinicians attributing higher weights to ‘impact on the patient’ (Additional file 1: Figure S1; scenario C-E). When applying the non-hierarchical weighting (not correcting for the number of criteria per group), the highest non-hierarchical weights were assigned to the individual criteria ‘case fatality ratio’, ‘spreading potential’ and ‘existing multidrug resistance’ (scenario G). The seed question for internal validity was answered consistently in 84% of responses (*n* = 67) and a scenario analysis was performed, which included only these consistent responses (scenario F).

### Scoring and ranking of diseases

**Fig. 4:**
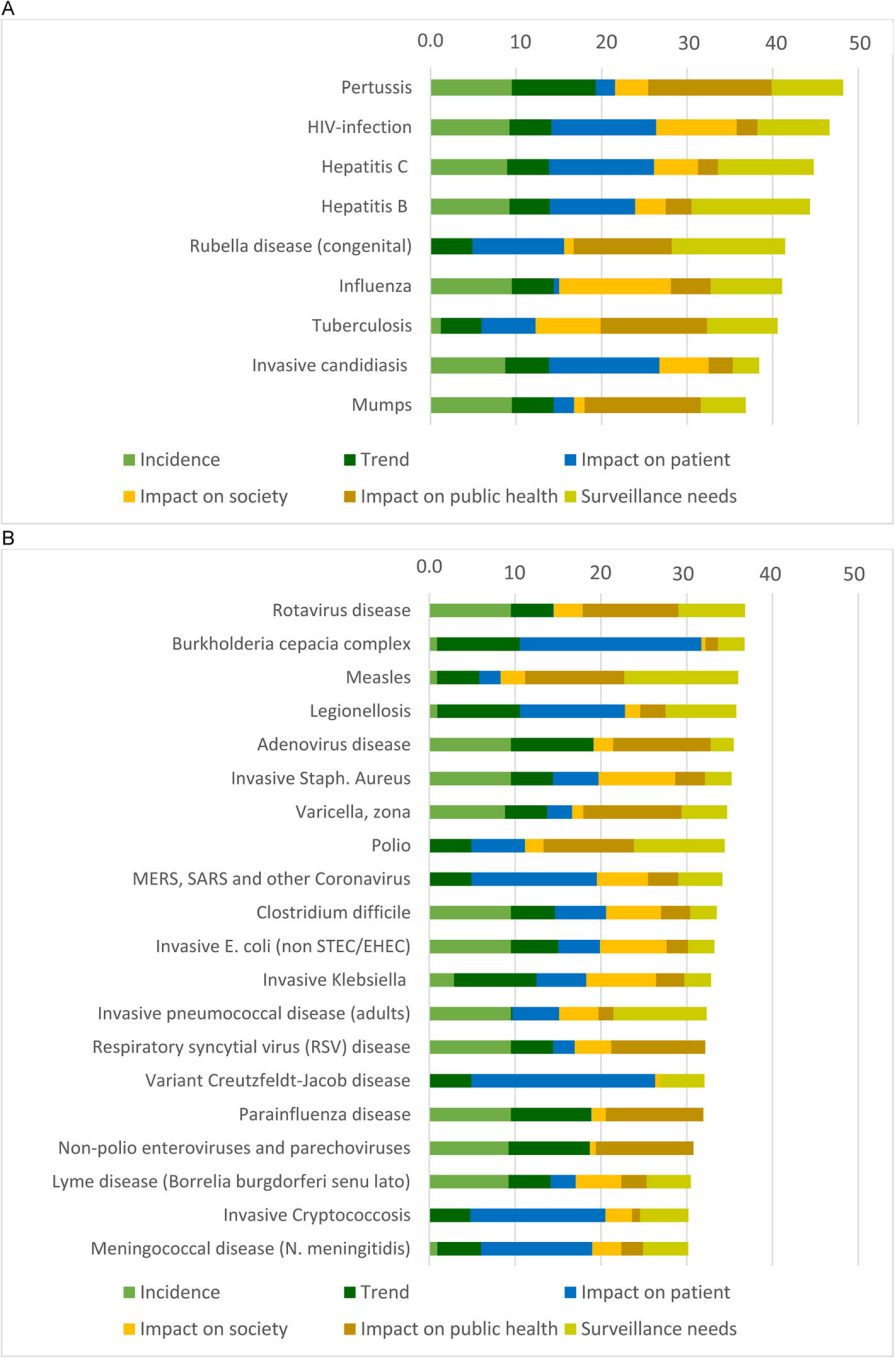
Composition of the final weighted score of the very high and high ranked diseases. Diseases in the very high (**a**) and high (**b**) priority groups are shown. The maximum possible score was 100. The score per criteria group was indicated by colours, and the criteria group incidence and trend was shown in two separate colours. Note that a default score of five points was obtained for the criteria ‘trend’ when no evidence for increasing or decreasing trends was observed

We received 37 responses in the second survey, concerning the scoring of the diseases against the criteria (14 microbiologists, 6 clinicians and 16 epidemiologists and 1 veterinarian). Based on the final weighted score per disease and the use of CART modelling [13] (Additional file 1: Figure S2), the diseases were classified in five priority groups: 9 diseases in the very high priority group, 20 in the high priority group, 28 in the medium priority group and, 21 in the low and 20 in the very low priority group (Additional file 1: Figure S3A, Table 2). The diseases with the highest rank were: pertussis, hepatitis C and hepatitis B and human immunodeficiency virus infection and the composition of the scores of all very high and high priority diseases was shown (Fig. 4). The composition of the scores for the remaining diseases was presented in the Suppl. information (Additional file 1: Figure S4). Pruning of the CART result with a complexity parameter of 0.05 (cp = 0.05), indicated that the least significant split was the one between the low and very low group, indicating that we may merge these two groups together into one low priority group (Additional file 1: Figure S2). An alternative classification, using visual inspection to define the priority groups, is shown (Additional file 1: Figure S3B).

**Table 2:** Classification of diseases and diagnoses in priority groups, according to the final weighted score, displayed in ranked order

Very high priority	High priority	Medium priority	Low priority	Very low priority
Score ≥ 36.8	≥30.0 and < 36.8	≥22.0 and < 30.0	≥17.0 and < 22.0	Score < 17.0
Pertussis	Rotavirus disease	Invasive pneumococcal disease (children)	Campylobacteriosis	Echinococcosis
HIV-infection	*Burkholderia cepacia* complex	*Haemophilus influenzae* (Hib)	Tick-borne encephalitis	Yersinia enterocolitica and pseudotuberculosis
Hepatitis C	Measles	Clostridium botulinum	Chlamydia pneumoniae	Vibrio cholerae and parahaemolyticus
Hepatitis B	Legionellosis	Helicobacter pylori	Zikavirus disease	West Nile virus disease
Rubella disease (congenital)	Adenovirus disease	Invasive Enterococci	Human Papillomavirus (HPV) infection	Parrot fever
Influenza	Invasive Staph. Aureus	*Neisseria gonorrhoeae*	Herpes simplex (CSF)	Rabies
Tuberculosis	Varicella, zona	Syfilis	Giardiasis	Hantaanvirus disease
Invasive candidiasis	Polio	Human metapneumovirus (HMPV)	Leishmaniasis	Smallpox
Mumps	MERS, SARS and other Coronavirus	Yellow fever disease	Invasive Aspergillosis	Rickettsia
	*Clostridium difficile*	Congenital CMV disease	Tularaemia (Francisella tularensis)	Ebola
	Invasive *E. coli* (non STEC/EHEC)	Congenital Toxoplasmose	Hepatitis E	Malaria
	Invasive Klebsiella	Tetanus	Trichinosis / trichinellosis	Anaplasmosis
	Invasive pneumococcal disease (adults)	Chikungunya disease	Congenital Parvovirus B19	Hepatitis A
	Respiratory syncytial virus (RSV) disease	Mycobacterium non-tuberculosis	Invasive Acinetobacter	Q-fever
	Variant Creutzfeldt-Jacob disease	Listeriosis	Invasive Strep. pyogenes (GAS)	Burkholderia mallei/pseudo mallei
	Parainfluenza disease	Shigellosis	Salmonellosis (Typhoid)	Amoebiasis
	Non-polio enteroviruses and parechoviruses	Cryptosporidiosis	Invasive and/or congenital Strep. agalactiae (GBS)	*Bacillus cereus*
	Lyme disease (Borrelia burgdorferi senu lato)	Chlamydia trachomatis	Brucellosis	Pasteurella
	Invasive Cryptococcosis	Pseudomonas	Dengue	Cyclospora
	Meningococcal disease (N. meningitidis)	Salmonellosis (non-typhoid)	Scabies	
		*Bacillus anthracis*	Bartonellosis (B. henselae)	
		Shiga-toxin producing E. coli		
		Chagas disease		
		Mycoplasma pneumoniae		
		Dyphtheria		
		Tropheryma whipplei		
		Leptospirose		
		Norovirus disease		

The five priority groups were defined by CART regression. The theoretical maximum possible score per disease was 100, the highest obtained score was 48 (Pertussis).

### Priorities per disease group and health domain perspectives

**Fig. 5:**
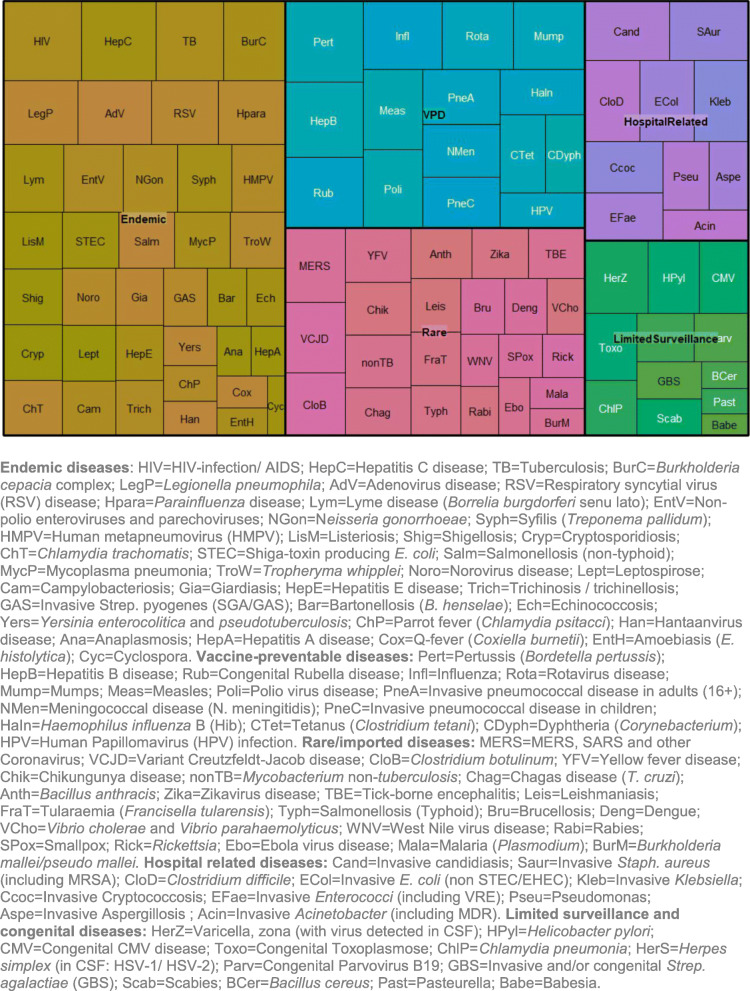
Treemap of the 98 diseases per thematic disease group. The size of the disease boxes represents the final weighted score per disease and main colours represent the thematic disease groups: vaccine-preventable diseases (*n* = 15), endemic diseases (*n* = 38), imported/rare diseases (*n* = 23), diseases with limited surveillance or congenital risks (*n* = 12), hospital-related diseases (*n* = 10)

**Fig. 6:**
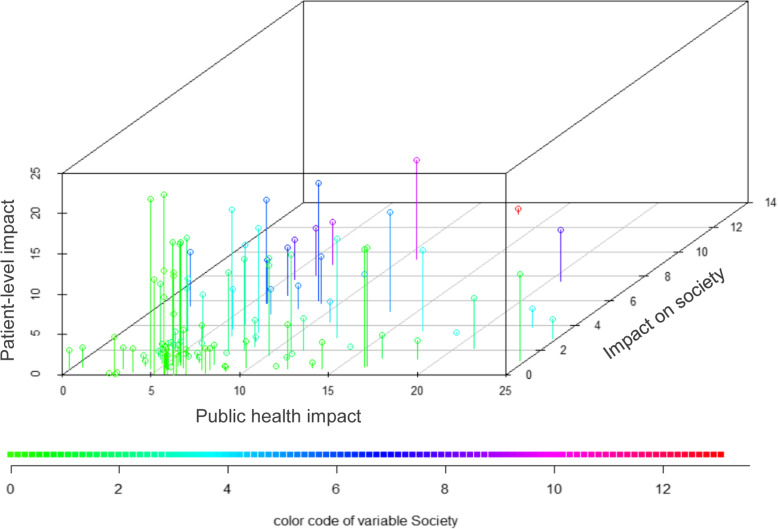
Three-dimensional plot of 98 diseases based on the weighted scores for the criteria groups patient-impact, society-impact and public health impact. The public health impact was here represented by the sum of ‘spreading potential’, ‘public health action required’ and the seven criteria of the criteria group ‘surveillance needs’. This plot provides insight in the priorities per health domain. Colours highlight the 3rd axes of the plot (society impact). This figure indicates that it is essential to appreciate the public health impact of diseases in parallel to the patient-level impact and the society impact of diseases

We compared the relative impact of the thematic disease groups, by visualising the final weighted scores per disease group using a treemap. This analysis identified the endemic diseases as the dominant disease group in our priority analysis (highest cumulative sum of final weighted scores), followed by the vaccine preventable diseases (Fig. 5). Besides comparing the final weighted score, we can compare disease based on the scores for the criteria groups patient-impact, society-impact and public health impact, which provides insight in the scatter of priorities per health domain. We plotted the public health impact versus the society-impact and the patient-level impact of the 98 diseases in a 3D plot, with colours highlighting the 3rd axes (Fig. 6). This figure indicate that from the ten diseases with a high public health score (≥15), only one had a moderate-high score for the society impact (≥6). Similarly, from the seven diseases with a moderate-high score for society impact (≥6), only one had a high score for public health impact (≥15), indicating it is essential to appreciate the public health impact of diseases separately from the society impact of diseases.

### Sensitivity analysis: alternative analysis scenarios

Eight alternative analysis scenarios were considered in order to assess the robustness of the ranking. The impact of the alternative analysis approaches on the composition of the top-20 ranked diseases was summarised (Additional file 1: Table 3, Figure S5). Taking into account experts opinions about ‘future risk’ introduced invasive *Klebsiella* infections into the top-20 ranked diseases (position 8), but the remaining composition of the top-20 did not change (scenario B). The use of alternative sets of weights resulted in a change of 0–2 diseases in the top-20 ranked diseases, except for the non-hierarchical set of weights (not account for the number of criteria within a criteria group; scenario G), which resulted in more changes. With this non-hierarchical interpretation of the weights, ‘case fatality ratio’, ‘spreading potential’ and ‘existing multi-drugs resistance’ ended up the highest and applying these weights to obtain the ranking, resulted in six replacements within the top-20 ranked diseases compared to the reference analysis (scenario G). The use of an alternative quantification of the answer categories (scenario H, Additional file 1: Table S1) resulted also in the replacement of six diseases compared to the reference scenario. The alternative quantification attributed higher values to the intermediate/neutral answer categories, except for the criteria ‘trend’, where neutral trends were devaluated and therefore more equal to diminishing trends (Additional file 1: Table S1). Interestingly, the diseases that entered the top-20 in at least two alternative scenarios, were all classified already in the very high or high priority group in the reference analysis (top-29 ranked diseases), except for pneumococcal disease in children and *Haemophilus influenza* infections; these two diseases may therefore also be considered high priority.

**Table 3:** Scenario analyses: eight alternative analysis scenarios and the resulting impact on the composition of the top-20 ranked diseases

ID	Study step	Scenario name	Description	Impact on top-20 priority classification^a^
A	–	Default	The default analysis scenario, including hierarchical weighting from all professions without future risks considered.	Reference
B	Criteria selection	Future risk	Accounts for the additional criterion ‘future risk’.	1/20
C	Weighting	Epidemiologists	Weighting according to profession: epidemiologists.	2/20
D	Weighting	Clinicians	Weighting according to profession: clinicians.	0/20
E	Weighting	Microbiologists	Weighting according to profession: microbiologists.	0/20
F	Weighting	Consistent respondents	Weighting that only takes into account responses with valid validation answers.	0/20
G	Weighting	Non-hierarchical Weighting	Non-hierarchical weighting: not accounting for the number of criteria within a criteria group.	6/20
H	Scoring	Quantification of answer categories	Alternative set of quantifications representing answer categories of the scoring survey (Table S1).	6/20

^a^Number of diseases originally found in the top-20 ranked diseases that got replaced in the alternative scenario.

### Principal component analysis and correlogram

**Fig. 7:**
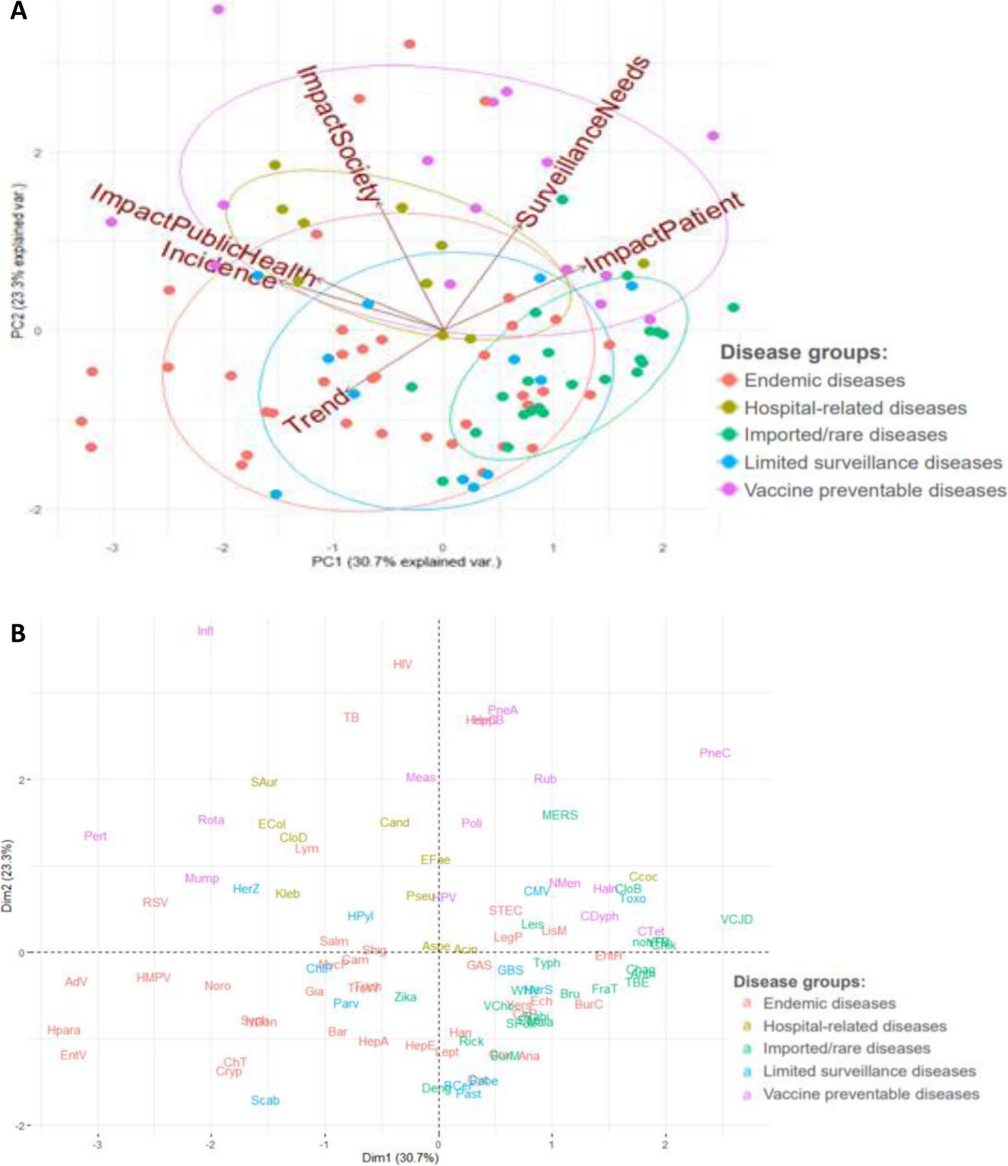
Principle component analysis (PCA) of the final weighted score, based on the weighted score of the six criteria groups for the 98 diseases. The orientation of the criteria groups are plotted as arrows on the two first principle components (**a**). The criteria ‘incidence’ and ‘trend’ are here considered as separate criteria groups. Each disease is represented by a dot (**a**) or an abbreviation of the disease name (**b**), of which the colours indicate the thematic disease groups. The scatter of the 98 diseases in the first two principle components shows clustering of the thematic disease groups. The classification of diseases in the thematic disease groups and disease labels are equal to that shown below Fig. 5

We analysed the composition of the final weighted score in further detail by a PCA, using the six criteria groups as loading factors (incidence and trend were used as two separate groups in this analysis), and obtained six principal components (PC). The first PC captured 31% of the variance, and the second captured an additional 23% (Additional file 1: Figure S6). ‘Incidence’ and ‘impact on the patient’ had the largest impact on the first component, whereas ‘impact on society’ and ‘surveillance needs’ contributed the most to the second component (Additional file 1: Figure S6). Plotting of the 98 diseases in these first two dimensions, showed a clustering of the diseases according to the thematic disease groups: for example endemic diseases tended to cluster in the left lower quadrant, while rare and important diseases tended to cluster in the right lower quadrant (Fig. 7). These examples show that the final weighted score of imported and rare diseases was positively determined by ‘impact on the patient’ and negatively by ‘incidence’, while the score of endemic diseases was highly positively determined by ‘incidence’ and ‘trend’. A similar clustering of the thematic disease groups was observed in the plot of the first two PC dimensions of the PCA based on the 18 individual criteria (Additional file 1: Figure S7). Further correlations between the 18 individual prioritisation criteria were analysed using a correlogram (Additional file 1: Figure S8). Numerous significant correlations (*p* < 0.05) were identified, showing for example that ‘incidence’ was positively correlated with ‘absenteeism’, ‘costs’ and ‘health care utilisation’, while it was negatively correlated with the prioritisation criteria ‘need of an NRC for the diagnosis’, ‘severity’ and ‘case fatality ratio’ (Additional file 1: Figure S8).

### Identification of uncertainties and data gaps

Disease groups with the highest number of blank answers were identified as the disease groups ‘limited surveillance and congenital diseases’ and ‘hospital-related diseases’ with a mean of 17 non-blank answers from 37 participants, compared to a mean of 20–24 non-blank responses for the other disease groups (Additional file 1: Table S2). The criteria with the highest number of blank answers were ‘events requiring public health action’, ‘healthcare utilisation’ and ‘excess costs’, with respectively a mean of 13, 14 and 18 non-blank answers (average of all diseases), compared to an average of 20–28 non-blank answers for the other criteria (Additional file 1: Table S2). Remarkably, the diseases identified with the highest standard error of the final weighted score were six hospital-related diseases, namely (in decreasing order, starting with the disease with highest standard error) invasive *Staphylococcus aureus*, invasive *Klebsiella*, invasive *Candida*, *Pseudomonas*, invasive *Enterococci* and *Clostridium difficile* disease, followed by *Influenza* (Additional file 1: Figure S3).

## Discussion

We ranked 98 infectious diseases according to the relative importance for surveillance and public health, based on 18 weighted prioritisation criteria, structured in five weighted criteria groups. Here, we discuss the impact of the used criteria set and the interpretations of the analysis scenarios. We interpret the resulting ranking in comparison to other disease ranking studies, like burden of disease studies. We analyse further strengths and limitations of our approach and provide recommendations for the implementation to inform future public health action in Belgium.

### Criteria correlations and scenario analyses

We designed a set of 18 prioritisation criteria, structured in five criteria groups. The correlogram showed that the set of criteria was balanced and well-chosen: none of the criteria showed an exact equal pattern of correlations, except absenteeism and cost, but we presume that this correlation was largely based on the lack of accurate data for both criteria. Therefore, none of the criteria were redundant.

We considered alternative analysis approaches as a sensitivity analysis. Four out of five scenarios with an alternative weighting showed to have no or minimal impact on the final composition of the top-20 ranked diseases, by an exchange of 0–2 diseases in the top-20 ranked diseases. We observed marked differences in the weighting of the criteria between different professional groups, as observed in previous studies [5], but this did not affect strongly the final composition of the top-20 ranked diseases. In contrast, the non-hierarchical interpretation of the weights showed to have considerable impact on the relative ranking (6/20 diseases in the top-20 ranked diseases got replaced). Note, however, that it is not valid to apply the non-hierarchical weights, as the survey and the composition of the criteria set were designed to calculate the weights in a hierarchical manner. This scenario was included because participants possibly could have misinterpreted the weighting exercise as non-hierarchical. The observed differences learn us that the design of the criteria set does partly determine the resulting relative ranking and, therefore, the criteria set and structure should be carefully designed.

The scenario including ‘future risk’ showed that few diseases obtained a higher ranking, but the overall ranking remained stable. The final alternative scenario touched on the topic of scaled values for the answer categories. We had attributed scaled values according to the scale of the answer categories (linear for linear scales, logarithmic for logarithmic scales), however, alternative -less intuitive- scales showed to impact the final ranking. Alternative scaling of the individual answer categories may be explored in further research, for example using conjoint analysis [14]. Overall, the scenario analyses showed that the composition of the very high and high priority group is fairly robust.

The definition of the priority groups that classify the diseases was objectified by using the CART regression tool [13]. The result of the CART classification were reassuring, as the resulting priority groups were fairly similar to those expected based on visual inspection. We choose to disclose also the exact relative ranking of the diseases within the priority groups, however, we recommend not to over-emphasize the relative position of diseases that were ranked within the same priority group. In addition, the sub-scores for the criteria groups were disclosed, which may resonate better with intuitive expectations than the final score only.

### Comparison with other disease ranking studies

We compared the ranks of the 29 diseases obtained in the prioritisation study with the ranks based on the DALY/100,000 population obtained in the EU burden of disease study [15], per disease group (Additional file 1: Figure S9). The prioritisation study did rank the vaccine preventable disease higher compared to the rank based on the DALY/100,000 population (except for *Influenza*, pneumococcal disease and *Haemophilus influenza*). In contrast, for most endemic diseases a lower rank was obtained in the prioritisation study compared to the rank based on DALY/100,000 population (except for gonococcal disease, syphilis and cryptosporidiosis). Burden of disease studies quantify the population impact of a disease by the population-sum of the patient-level impact. However, the MCDA approach shows that the impact of a disease on the society and on public health are additional parameters to take into account on top of the patient-level impact. Therefore, the cumulative patient-level impact resulting from burden of disease studies is not fully reflecting the impact of a disease in the population, because important additional perspectives (society and public health impact) are missing. Our study showed that the highest weights were assigned to public health parameters, which were thus considered the most important factors for priority setting of communicable diseases in Belgium. Future studies may focus on a more detailed quantification of these public health parameters for the individual diseases. It should be recognise that priorities for public health may differ from priorities in daily disease management, in order to allow differentiation of priorities for both health domains.

Furthermore, we compared our ranking results with existing literature reporting the priority ranking of infectious diseases in other countries (Additional file 1: Figure S10) [5]. Our study ranked vaccine preventable diseases higher compared to the German study of 2011 (except for HPV and pneumococcal disease, which were ranked higher in the German study; and hepatitis B and *Neisseria meningitides*, which were ranked almost equal in both studies). Most endemic diseases were ranked similarly in both studies. Disease that were ranked notably high in the German study, but not in our study were: campylobacteriose, group A streptococcal disease and Hantavirus. Disease that were ranked notably high in our study, but not in the German study were for example: pertussis, rubella, Lyme disease and *Shigella*. Differences may be explained by differences in the countries epidemiology and context, but may also be explained by methodological choices.

Furthermore, it is notable that some diseases, for example *Burkholderia* and Chagas disease, with a major impact on the patient but among a limited risk group only (cystic fibrosis or sickle cell anaemia in case of *Burkholderia*; ethnic minority in case of Chagas disease) are ranked relatively high in our prioritisation approach compared to other ranking studies and burden of disease studies. A similar remark can be made concerning diseases with no or very rare occurrence in our country: they may be ranked high based on a potential high patient-level impact, but this is an overestimation of its real impact as no cases are occurring in our country. We should therefore recognise that our study focussed on priority ranking of infectious diseases that are occurring in our country; the risk ranking of emerging diseases would need another approach and would require different sets of criteria and weightings.

### Strengths of the used approach

The strengths of our approach are mainly the transparent and standardised MCDA methodology. We have further objectified the methodology by selecting quantitative variables, which allowed to implement disease-specific surveillance data and evidence in our analysis. For the qualitative criteria (based on expert opinion), we collected evidence-based help information (fact sheets). This help information also compensated for an potential lack of ready knowledge of the experts for all 98 diseases. The experts were encouraged to consult this information for attributing their scores to the criteria. Experts were able to restrain from answering each individual question or disease, when they did not feel confident to answer. Although the consultation of expert opinions introduced some subjectivity, the consultation of the whole panel of experts resulted in a dilution of the effect of individual subjectivity. Our combined approach, based on both quantitative (evidence-based data) and qualitative criteria (based on expert opinion), was able to balance the limitations encountered in full quantitative or full qualitative studies, such as the lack of data (quantitative methods) or the risk for subjectivity and unreliability (qualitative methods). A strict quantitative approach can only be applied to a restricted number of diseases and criteria for which an exhaustive quantitative database is available, and therefore currently cannot cover all important aspects of infectious disease priority ranking.

### Validity of our study

The MCDA methodology is recommended for the comprehensive risk ranking of infectious diseases, including novel, emerging and established infections. We clearly described how criteria were chosen. The criteria covered all of the key communicable disease facets (likelihood, how easily the disease could be spread, the need for public health measures, impact on school and work absenteeism, severity and duration of illness, diagnostic issues and preventive measures currently in place (e.g. vaccinations). Acceptability and intelligibility of the surveys were tested by piloting of the surveys by multiple persons with different professional background; followed by small adaptations of the survey to improve both facets. Internal consistency of the survey that collected the weights of the criteria was tested by asking participants a repeated question in a variant form. Interrater variation was reduced by providing full criteria and scoring definitions within both surveys. Validity of the ‘fact sheets’ accompanying the second survey was ensured by consulting minimal two disease-specific experts who had to agree in case of initial disconcordance. Validity of the participant responses in the second survey was increased by allowing respondents to acknowledge the limits of their knowledge (skip complete disease groups, or to answer ‘I have no opinion’ for individual questions and diseases). The validity of the final priority classification was tested in a sensitivity analysis that showed the impact of assumptions made in the different analysis scenario’s. We included tests to measure variation between professional groups (analysis scenario C, D, E).

### Limitations of the used approach

We followed the recommendations for best practice in disease ranking projects [7], nevertheless, some limitations are inherent to our study. Selection bias of the participating experts may be present, because participation was anonymous and voluntary, however, we showed in the scenario analysis that the impact of professional background on the final resulting ranking was negligible.

A known limitation, inherent to any impact analysis of diseases for which successful control measures have been established, is the underestimation of vaccine-preventable diseases in standardised disease ranking studies. It remains difficult to account appropriately for the decreasing trend of vaccine-preventable diseases, following successful implementation of effective prevention programs. We accounted for this by including criteria such as ‘vaccine included in national vaccination program’ and ‘risk for vaccine triggered strain replacement’. Indeed, we observed that six of the nine diseases classified in the very high priority group in our study were vaccine-preventable diseases. Similarly, our study ranked vaccine preventable disease higher compared to both the EU burden of diseases study [15] and the German prioritisation study [5], suggesting that we effectively compensated for the underestimation of vaccine-preventable diseases. However, still some vaccine-preventable diseases might be ranked lower due to the success of control measures, which might be also the case for other diseases, like diseases provoked by multi-drugs resistant (MDR) organism, for which an increasing number of preventive measures are being put in place.

Another limitation is that the set of prioritisation criteria are not completely standardised across countries and studies. On the other hand, this provides the MCDA method its flexibility to adapt to contextual settings and objectives. However, the selected set of criteria and its structure may influence the final ranking. The scenario analysis indeed showed that the selected hierarchical structure of the criteria, may have influenced the final ranking result, because criteria in criteria groups with only a few criteria received higher weights for the individual criteria. On the other hand, the hierarchical structure in five main criteria groups, circumvented disadvantages inherent to an extended number of criteria.

Factors left outside the scope of this analysis form another limitation and concern for example ‘inequalities in disease distribution’, ‘potential for prevention and/or treatment/control’ (although partly covered by the criteria ‘proportion of events that require public health action’) and whether the disease was ‘acquired abroad’ or whether transmission was observed inside our country. The lack of consideration of the transmission chain may justify to argue that the rank of some rare and/or imported diseases may have been overestimated (for example this can be argued for tuberculosis). Separate consideration of rare/imported disease may facilitate the interpretation.

A general limitation to consider is that additive MCDA models do not account for interaction between the individual criteria. In other words, we did not ask the perceived relative weight of each answer category and we did not ask the weight of an answer category given an certain value for another criteria (for example the weight of high severity given a disease without occurrence). This could have been achieved by conjoint analysis, and would have informed the scaled values, but would have been much more time consuming for both investigators and voluntary participants to the expert panel.

A final limitation of our study is the emphasis of pathogen-specific diseases, as opposed to public health programs targeted to population groups rather than diseases (children, vulnerable adults, ..) or targeted to a group of diseases (like MDR organisms, nosocomial infections or sexual transmittable infections).

Although the MCDA ranking methodology reduces most subjectivity, some limitations remain inherent to its approach. Therefore, it might be of added value to search structured feedback of experts on the current obtained ranking in order to refine and consolidate its conclusions and explore its boundaries.

### Implementation in the Belgian context

The objective of this study was to inform the Belgian policy makers and managers, and we believe that our study has established an excellent basis to guide policy decisions. In Belgium, the infectious disease prevention programs and the list of diseases under mandatory notification are under regional control. The need for a prioritisation study has been raised by the regional health authorities, therefore we feel that there is a will among policy makers to use these priority ranking results and to apply an evidence based approach to prioritise resources.

In addition, our results may be used to review existing surveillance programmes in light of the obtained priority ranking. Currently, Belgium has a network of 41 national reference laboratories (NRC) and the list of diseases under surveillance should be reviewed recurrently by its steering committee, according to the legal law which institutionalised these centres [16]. The final priority score or individual prioritisation criteria are excellent objective parameters to gain inside in surveillance needs and priorities for NRCs.

Finally, primary care by general practitioners constitute an important pillar of health care in Belgium and fulfils an important first line function towards risk populations. Preventive public health opportunities in primary care are increasingly recognised, and the establishment of a priorities among communicable diseases within our study may help to implement focused public health services for patients at risk in primary care.

Our study showed that pertussis, HIV, hepatitis C and B are ranked as highest priority communicable diseases in Belgium. These diseases are included in current public health and surveillance programs, but additional programs among risk groups may be of public health value. Further exploration of the specific needs in Belgium concerning the very high and high priority communicable diseases is highly recommended. In order to explore the full opportunities of the ranking results revealed by our study, we recommend further cross-reference of the obtained ranking with current and planned public health and surveillance programs, to identify potential mismatched priorities. Furthermore, we recommend to implement this prioritization approach in a recurrent evaluation cycle of national public health priorities, in order to include newly emerging diseases and to identify shifted priorities over time.

## Conclusion

This project effectively ranked infectious diseases, based on a context specific quantitative variable and expert based weighting of these variables. The diseases ranked highest are included in current public health programs, but additional programs among risk groups might be valuable. Further cross-reference of the obtained ranking with current programs is recommended. We recommend to implement this method in a recurrent evaluation cycle of national public health priorities.

## Supplementary information

**Additional file 1 Table S1**: Answer categories and their scaling. **Figure S1**: Median weights of the 18 individual criteria per profession. **Figure S2**: CART decision tree. **Figure S3**: Definition of priority groups (A) by CART methods and (B) by visual inspection. **Figure S4**: Composition of the final score for diseases. **Figure S5**: Bump chart with ranking of diseases, comparing the analysis scenarios. **Figure S6**: PCA loading plots. **Figure S7**: PCA based on the 18 individual criteria, with the 98 diseases plotted. **Figure S8**: Correlogram of the 18 individual criteria, based on the 98 diseases. **Table S2**: Number of non-blank responses per individual criteria and per disease group. **Figure S9**: Comparison with burden of disease study 2018. **Figure S10**: Comparison with German prioritisation study 2011

**Additional file 2:.** “Survey for weights”.

**Additional file 3:.** “Survey for scores”.

## Data Availability

All data is included in the manuscript and the datasets generated and analyzed during the current study are available from the corresponding author on reasonable request.

## Abbreviations

BVIKM: Belgian Society of Infection Specialists and Clinical Microbiologists
CART: Classification and Regression Tree
CMV: Cytomegalovirus
CSF: Cerebrospinal fluid
DALY: Disease adjusted life years
ECDC: European Centre of Disease Prevention and Control
EHEC: Enterohemorrhagic *E. coli*
EU: European Union
GAS: Group A *Streptococcus*
GBS: Group B *Streptococcus*
HiB: *Haemophilus influenza* type b
HIV: Human Immunodeficiency Viruses
HPV: Human Papillomavirus
MCDA: Multi-criteria decision analysis
MDR: Multi-Drugs Resistance / Resistant
MERS: Middle-East Respiratory Syndrome
NRC: National Reference Centre
OIE: World Organization for Animal Health
PC: Principle Component
PCA: Principle Component Analysis
PH: Public Health
RSV: Respiratory syncytial virus
SARS: Severe acute respiratory syndrome
STEC: Shiga-toxin producing *E. coli*
WHO: World Health Organization
