# Supplementary material for: Prioritisation for future surveillance, prevention and control of 98 communicable diseases in Belgium: a 2018 multi-criteria decision analysis study
Source: BMC Public Health. 2021 Jan 22;21:192. doi: 10.1186/s12889-020-09566-9 (PMC7820105; doi:10.1186/s12889-020-09566-9)
Supplement: Supplementary file 1 — Additional file 1 Table S1: Answer categories and their scaling. Figure S1: Median weights of the 18 individual criteria per profession. Figure S2: CART decision tree. Figure S3: Definition of priority groups (A) by CART methods and (B) by visual inspection. Figure S4: Composition of the final score for diseases. Figure S5: Bump chart with ranking of diseases, comparing the analysis scenarios. Figure S6: PCA loading plots. Figure S7: PCA based on the 18 individual criteria, with the 98 diseases plotted. Figure S8: Correlogram of the 18 individual criteria, based on the 98 diseases. Table S2: Number of non-blank responses per individual criteria and per disease group. Figure S9: Comparison with burden of disease study 2018. Figure S10: Comparison with German prioritisation study 2011 [file 12889_2020_9566_MOESM1_ESM.pdf]

Table S1: Scaling of answer categories in the reference scenario A and alternative scenario H.

Criteria	Answer Categories	Scaled values Default Scenario A	Scaled values Alternative Scenario H
INCIDENCE	Rare	0.000	0
	Low	0.010	0.25
	Moderate	0.100	0.5
	High	1.000	1
TREND	Decline	0.000	0
	No apparent increase or decline	0.500	0.1
	Increase	1.000	1
CASE_FATALITY_RATIO	Rare	0.000	0
	Low	0.010	0.25
	Moderate	0.100	0.5
	High	1.000	1
SEVERITY	Very mild – Mild (moderate-severe cases may occur rarely)	0.000	0
	Mild – Moderate (severe cases may occur rarely)	0.330	0.25
	Moderate – Severe	0.660	0.5
	Mostly Severe	1.000	1
CHRONICITY	Low	0.000	0
	Moderate	0.100	0.5
	High	1.000	1
PH_ACTIONS	Small	0.000	0
	Moderate	0.500	0.5
	Large	1.000	1
SPREAD	Low	0.000	0
	Moderate	0.100	0.5
	High	1.000	1
ABSENTEEISM	Low	0.000	0
	Moderate	0.100	0.5
	High	1.000	1
HEALTH_CARE_UTILIZATION	Low	0.000	0
	Moderate	0.100	0.5
	High	1.000	1
EXCESS_COSTS	Low	0.000	0
	Moderate	0.100	0.5
	High	1.000	1
PUBLIC_ATTENTION	Low	0.000	0
	Moderate	0.100	0.5
	High	1.000	1
INTERNATIONAL_OBLIGATIONS	Yes	1.000	1.000
	No	0.000	0.000
WHO_OBJECTIVE	Yes	1.000	1.000
	No	0.000	0.000
VACCINE_IN_NVP	Yes	1.000	1.000
	No	0.000	0.000
VACCINE_TRIGGERED_REPLACEMENT	Yes	1.000	1.000
	No	0.000	0.000
EXISTING_MDR	Yes	1.000	1.000
	No	0.000	0.000
NRC_FOR_DIAGNOSIS	Yes	1.000	1.000
	No	0.000	0.000
CONGENITAL_RISKS	Yes	1.000	1.000
	No	0.000	0.000
FUTURE_IMPACT	Very low	0.00	NA
	Low	0.01	NA
	Medium	0.10	NA
	High	1.00	NA

Figure S1: Hierarchical weights of the 18 individual criteria per professional background. Weights are presented relatively to the criteria that was attributed the highest median weight while considering all experts ('spreading potential'). Colours represent professional background of the respondents: clinicians (n=18), epidemiologists (n=17) and microbiologists (n=35). Respondents who answered to have another professional background (n=10) were left out of this analysis.

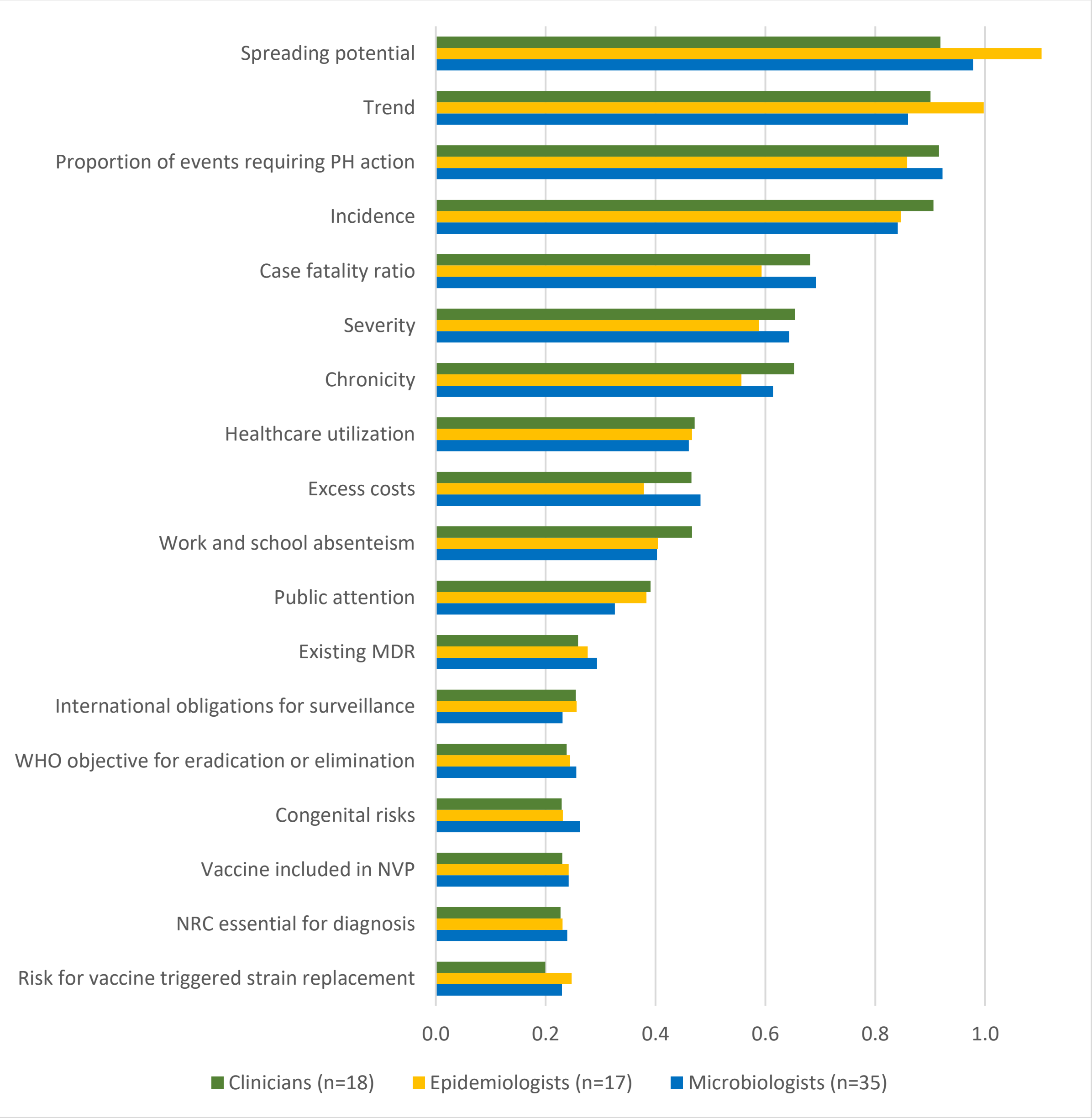

Figure S2: CART decision tree to define priority groups, weighted (A) and the same decision tree, but pruned (B).

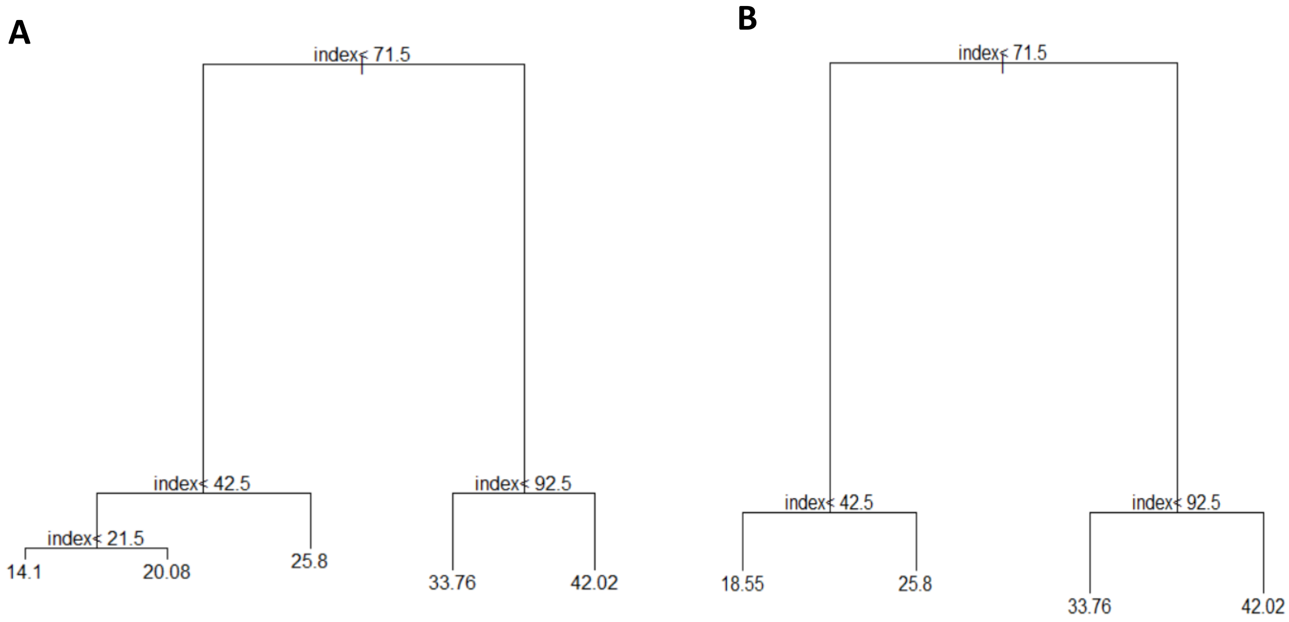

Figure S3: Final weighted scores (dots) and their standard errors (vertical bars), showing the definition of (A) the five priority groups based on CART regression and (B) the four priority groups based on visual inspection. Maximum possible score was 100.

A

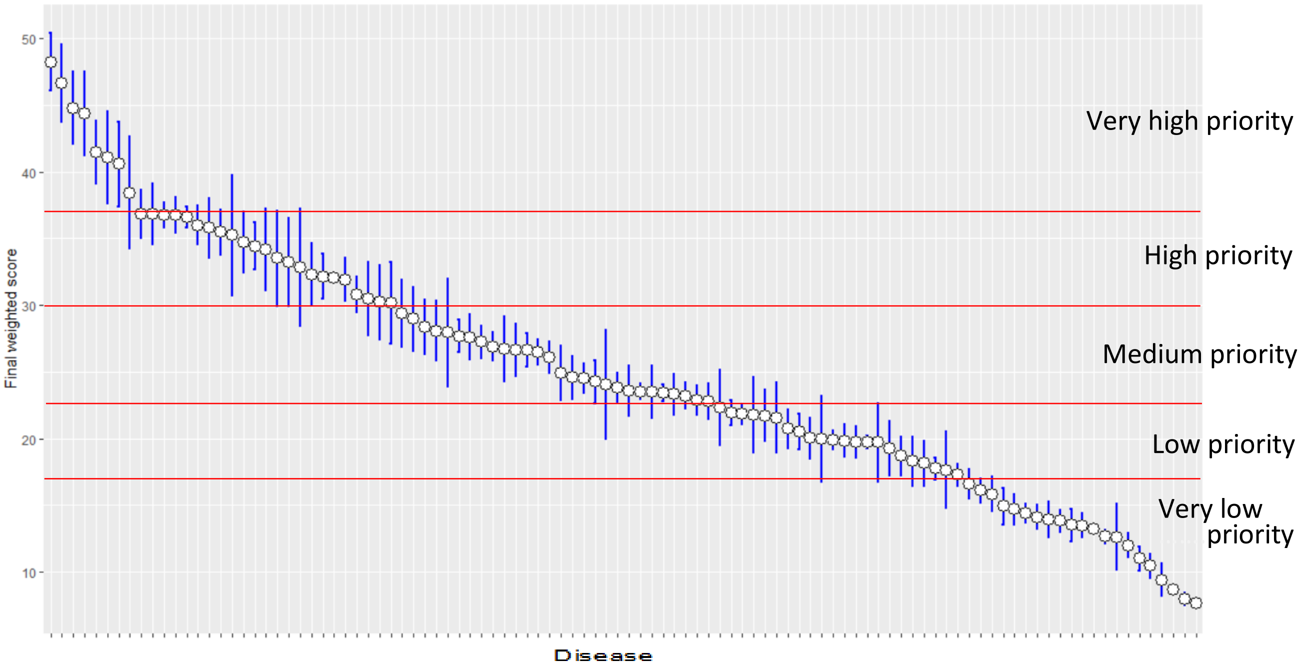

B

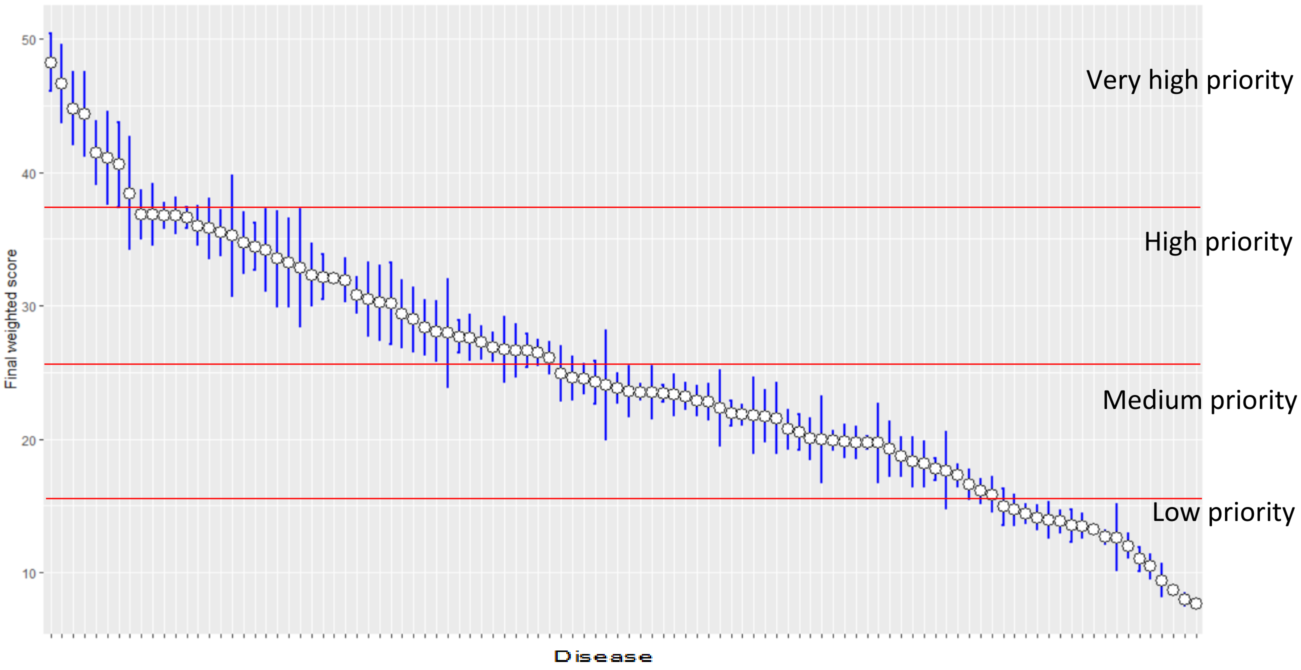

Figure S4: Composition of the final weighted score for diseases classified as medium priority (A), low priority (B) and very low priority (C). The theoretical maximum possible score was 100.

A:

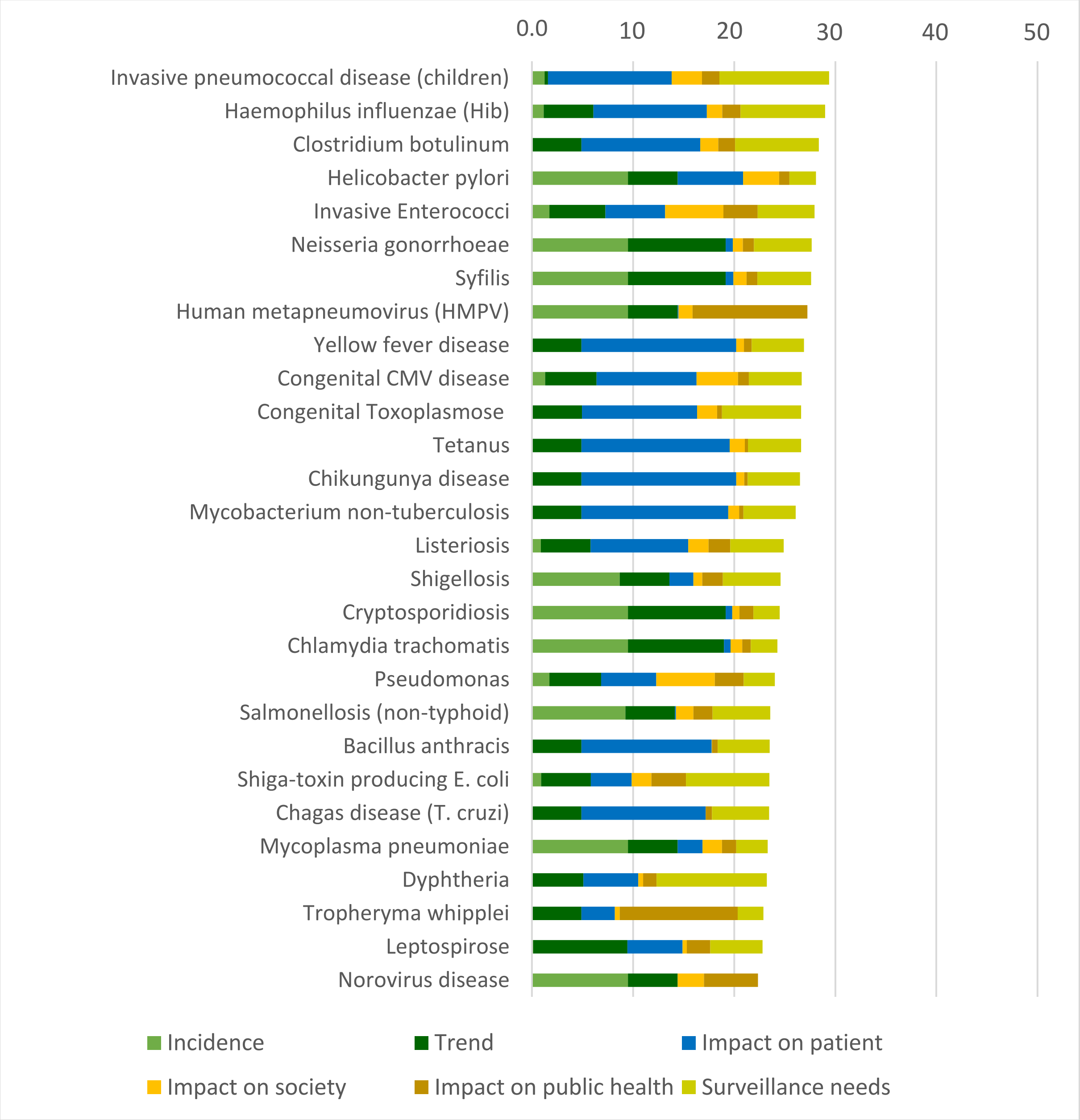

B:

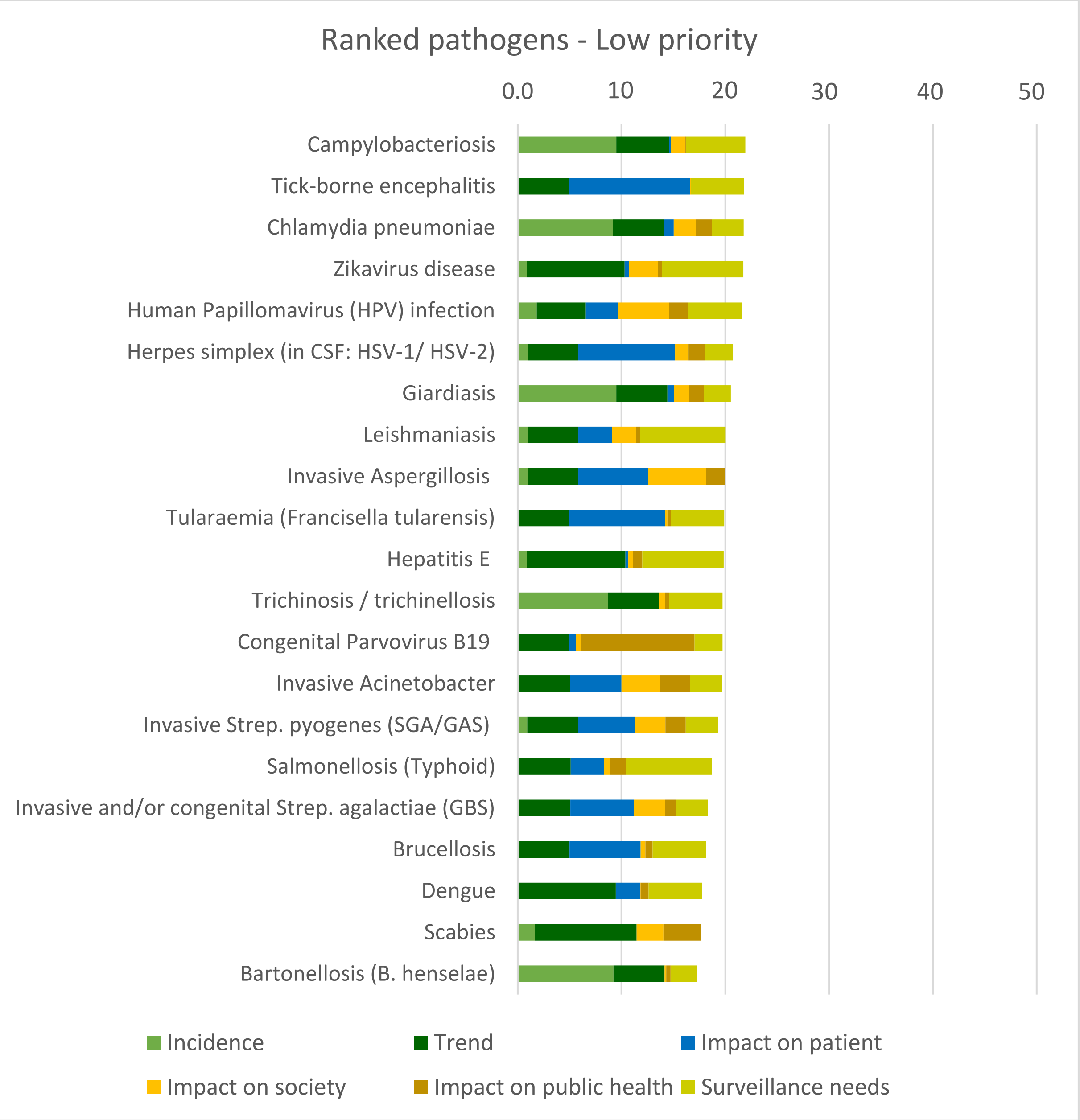
Ranked pathogens - Low priority
0.0
10
20
30
40
50
Campylobacteriosis
Tick-borne encephalitis
Chlamydia pneumoniae
Zikavirus disease
Human Papillomavirus (HPV) infection
Herpes simplex (in CSF: HSV-1/ HSV-2)
Giardiasis
Leishmaniasis
Invasive Aspergillosis
Tularaemia (Francisella tularensis)
Hepatitis E
Trichinosis / trichinellosis
Congenital Parvovirus B19
Invasive Acinetobacter
Invasive Strep. pyogenes (SGA/GAS)
Salmonellosis (Typhoid)
Invasive and/or congenital Strep. agalactiae (GBS)
Brucellosis
Dengue
Scabies
Bartonellosis (B. henselae)
Incidence
Trend
Impact on patient
Impact on society
Impact on public health
Surveillance needs

C:

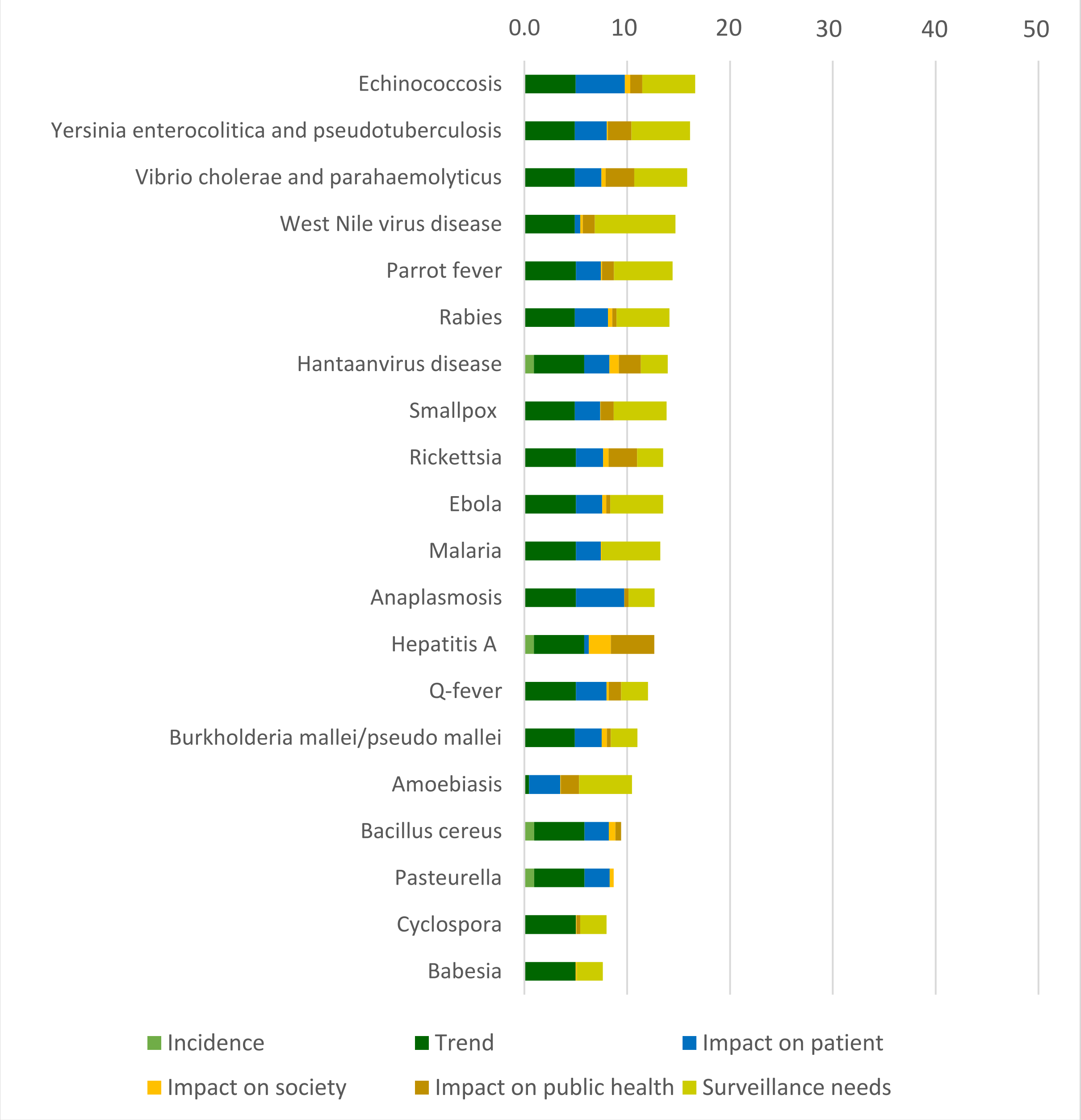
0.0
10
20
30
40
50
Echinococcosis
Yersinia enterocolitica and pseudotuberculosis
Vibrio cholerae and parahaemolyticus
West Nile virus disease
Parrot fever
Rabies
Hantaanvirus disease
Smallpox
Rickettsia
Ebola
Malaria
Anaplasmosis
Hepatitis A
Q-fever
Burkholderia mallei/pseudo mallei
Amoebiasis
Bacillus cereus
Pasteurella
Cyclospora
Babesia
Incidence
Trend
Impact on patient
Impact on society
Impact on public health
Surveillance needs

Figure S5: Composition of the top-20 ranked diseases in each of the scenario analyses. Numbers refer to the ranking, colours refer to diseases. The legend of diseases is displayed in order of the ranking in the reference scenario (Scenario A). Grey colours indicate newly introduced diseases into the top-20 in comparison to the reference scenario (Scenario A).

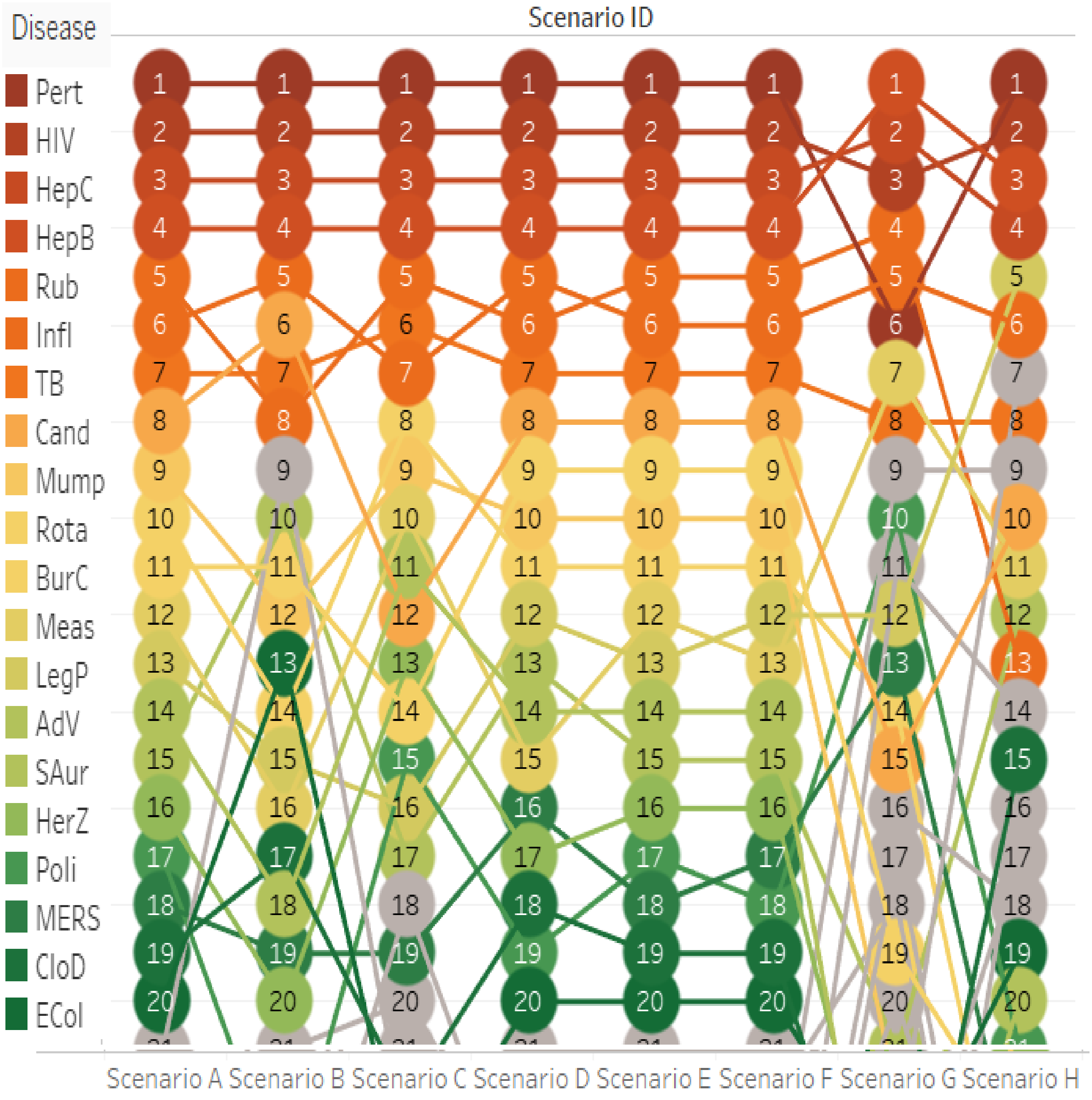

Disease legend: Pert=Pertussis (*Bordetella pertussis*); HIV=HIV-infection/ AIDS; HepC=Hepatitis C disease; HepB=Hepatitis B disease; Rub=Congenital Rubella disease; TB=Tuberculosis; Infl=Influenza; Cand=Invasive candidiasis; Rota=Rotavirus disease; BurC=*Burkholderia cepacia* complex; Mump=Mumps; Meas=Measles; LegP=*Legionella pneumophila*; AdV=Adenovirus disease; Saur=Invasive *Staph. aureus* (including MRSA); HerZ=Varicella, zona (with virus detected in CSF); Poli=Polio virus disease; MERS=MERS, SARS and other Coronavirus; CloD=*Clostridium difficile*; ECol=Invasive *E. coli* (non STEC/EHEC).
Legend of grey diseases: Scenario B, rank 9= Invasive *Klebsiella*; Scenario C, rank 18= Human Parainfluenza disease; rank 20= Respiratory syncytial virus (RSV) disease; Scenario G, rank 9= Invasive pneumococcal disease in adults (16+); rank 11= Invasive pneumococcal disease in children; rank 16= *Haemophilus influenzae* B (Hib); rank 17= *Clostridium botulinum*, rank 18= Variant Creutzfeldt-Jacob disease; rank 20= Invasive Cryptococcosis; Scenario H, rank 7= Invasive *Klebsiella*; rank 9= Invasive pneumococcal disease in adults (16+); rank 14= Invasive pneumococcal disease in children; rank 16= Non-polio enteroviruses and parechoviruses; rank 17= Invasive Enterococci (including VRE); rank 18= *Haemophilus influenzae* B (Hib).

Figure S6: Principle component analysis (PCA) of the final weighted score, based on the weighted score of (A, B) the six criteria groups and (C, D) the 18 individual criteria for the 98 diseases. 'Trend' and 'incidence' were analysed as separate criteria group. The criteria (groups) are plotted as arrows on the two principle components that explain the highest proportion of the total variance, with the length of the arrow and the colours indicating the relative contribution of the separate criteria groups to these components (red=high; yellow= intermediate; blue=low) (A, C). The loading plots for the first and second component, showing the contribution of the separate criteria groups to these components, with colours indicating the direction of the contribution (green=positive; red=negative) (B, D).

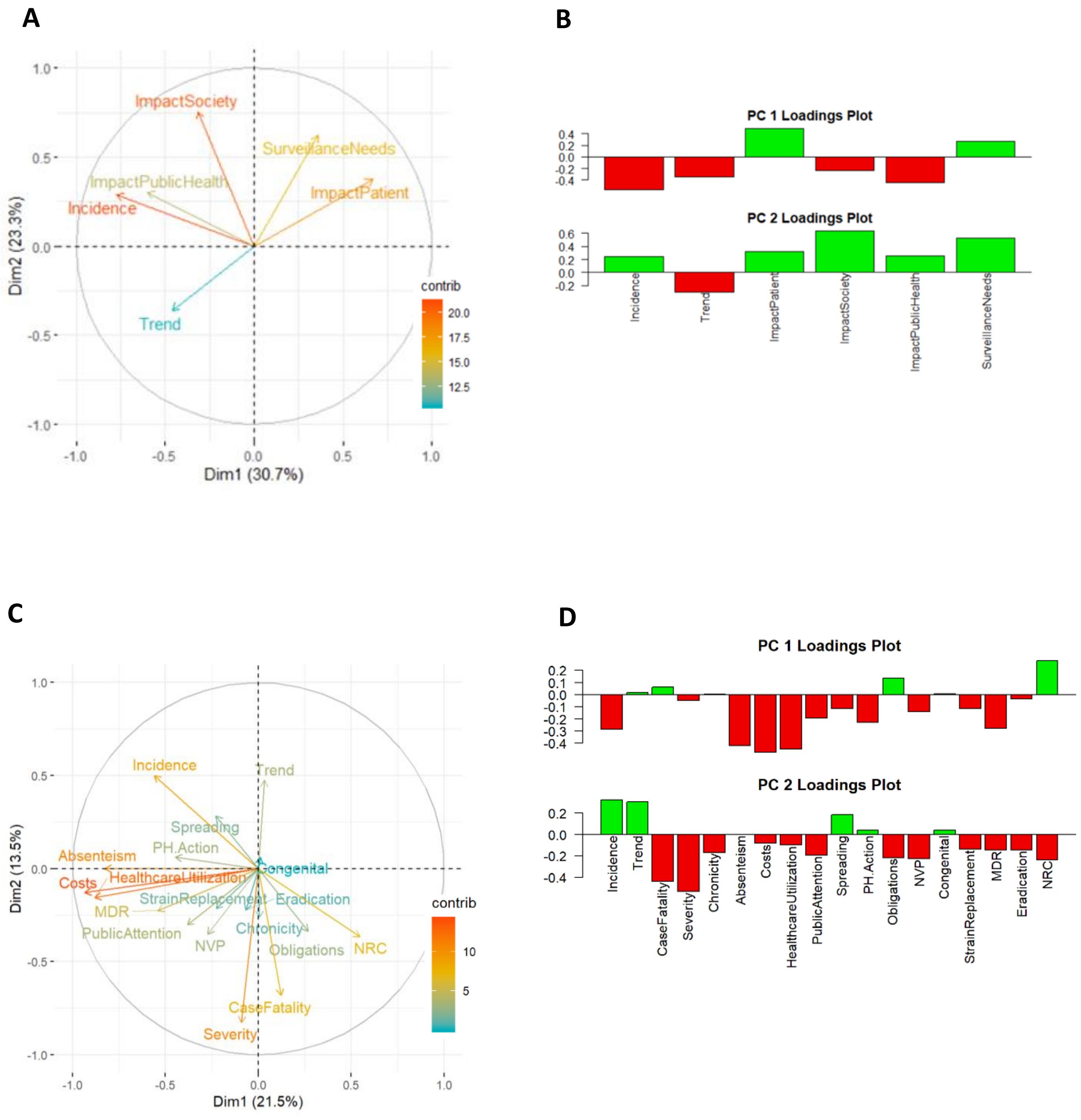

Figure S7: Principle component analysis (PCA) of the final weighted score, based on the weighted score of the 18 individual criteria for the 98 diseases. The orientation of the criteria are plotted as arrows on the two first principle components (A). Each disease is represented by a dot (A) or an abbreviation of the disease name (B), of which the colours indicate the thematic disease groups. The scatter of the 98 diseases in the first two principle components shows clustering of the thematic disease groups. The clustering of disease groups here is more pronounced than based on the criteria group PCA (Figure 7), but a lower percentage of variance is explained. The classification of diseases in the thematic disease groups and disease labels are equal to that shown below Figure 5.

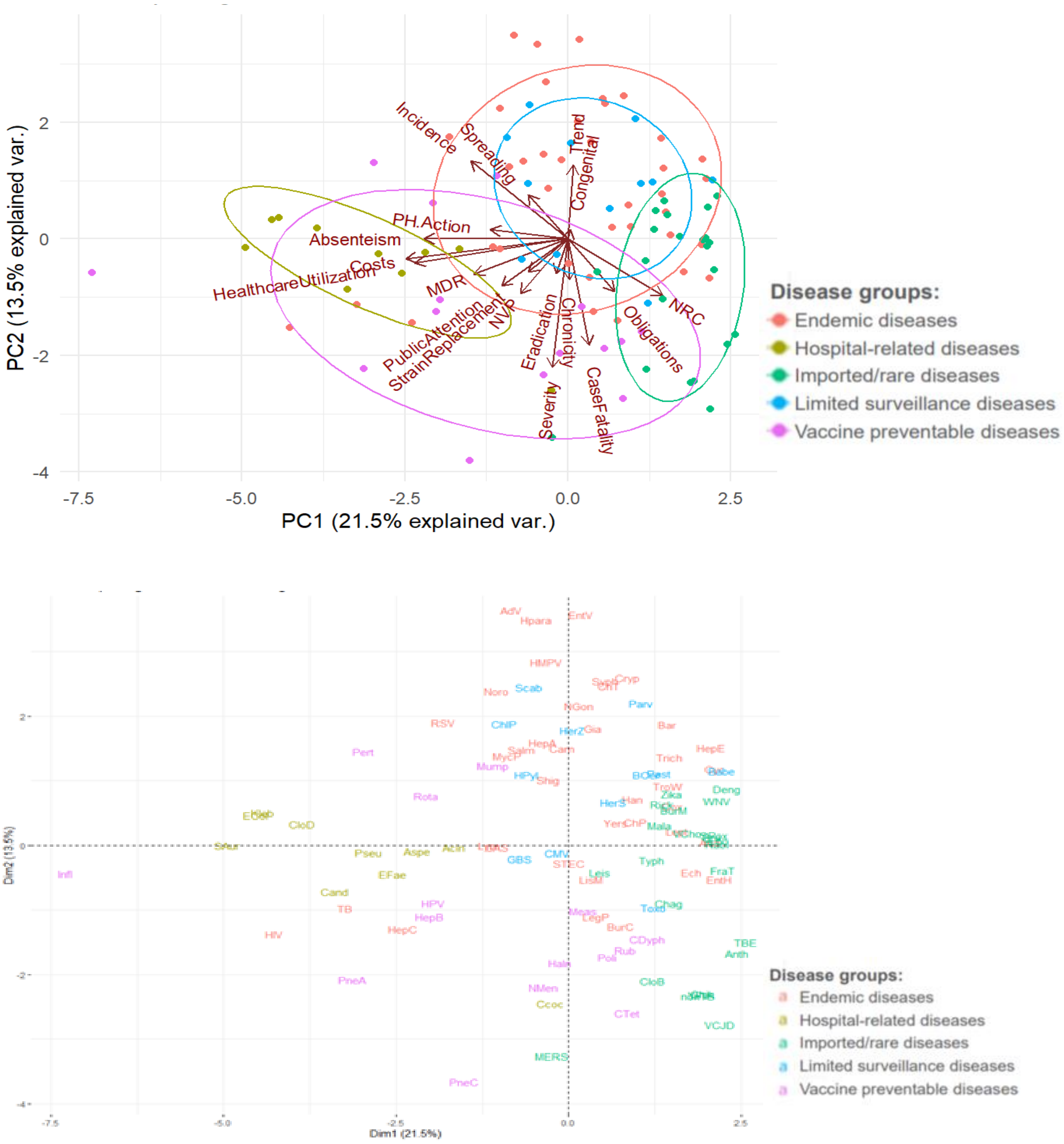

Figure S8: Correlogram based on the weighted score of the 18 individual prioritisation criteria for the 98 diseases. Colours represent strength and direction of the correlation (red=negative correlation; blue=positive correlation). Significance was considered when p<0.05. Non-significant correlations are left blank.

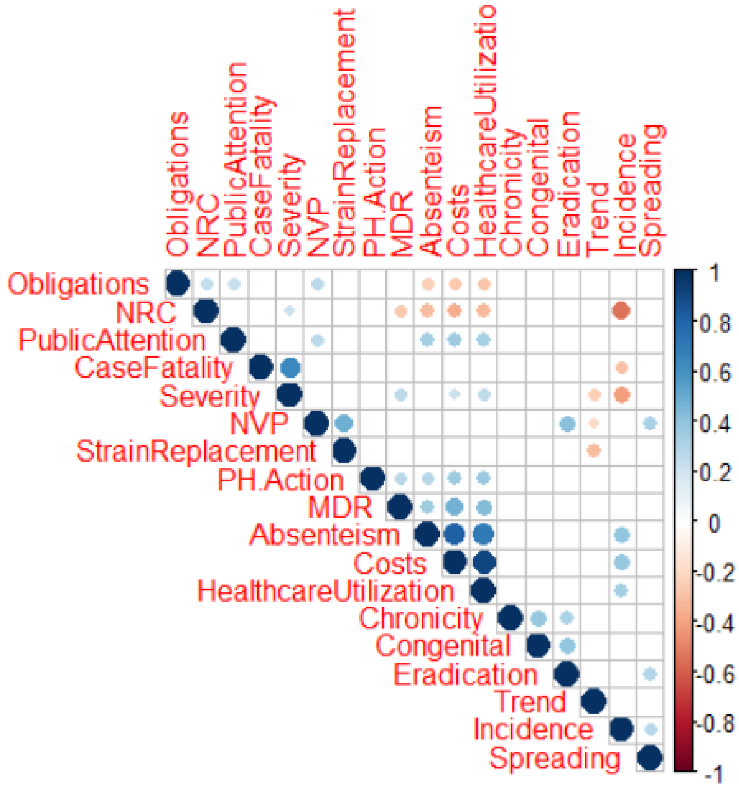

Table S2: Number of non-blank responses per criteria and disease in the scoring survey. In total 37 respondents participated in the survey. Respondents had the possibility to skip questions for whole thematic disease groups per criterion. The classification of diseases in the thematic disease groups is equal to that shown below Figure 5.

	Mean non-blank responses per disease group					
	Vaccine-preventable	Endemic	Rare, Imported	Limited surveillance	Hospital-related	Overall
**Incidence**	30	31	28	25	22	**28**
**Trend**	28	29	26	24	23	**27**
**Case fatality ratio**	24	25	20	18	16	**22**
**Severity**	24	26	21	16	15	**22**
**Chronicity**	23	23	18	18	14	**20**
**Work and school absenteeism**	22	22	20	14	16	**20**
**Excess costs**	21	19	17	14	14	**18**
**Healthcare utilization**	17	16	12	9	12	**14**
**Public attention**	29	27	25	22	23	**26**
**Spreading potential**	24	24	17	17	18	**21**
**Proportion of events requiring PH action**	15	13	12	10	11	**13**
**Future risk**	27	22	22	20	23	**22**
**Overall**	**24**	**23**	**20**	**17**	**17**	**21**

Figure S9: Ranks of 29 diseases included in both our prioritisation study and the EU Burden of disease study (Cassini, 2018). We compared per disease group our ranking with the ranks based on the DALY/100 000 population obtained by Cassini *et al*. Note that axes are shown in reverse order, visually emphasizing that lower ranks represent higher priority. In general, our prioritisation study obtained lower ranks (higher priority) for the vaccine preventable diseases compared to the ranks based on the DALY/100 000 population (except for *Influenza*, pneumococcal disease and *Haemophilus influenza*). In contrast, for the endemic diseases often lower priority (higher ranks) were obtained in our prioritisation study compared to the rank based on DALY/100 000 population (except for gonococcal disease, syphilis and cryptosporidiosis).

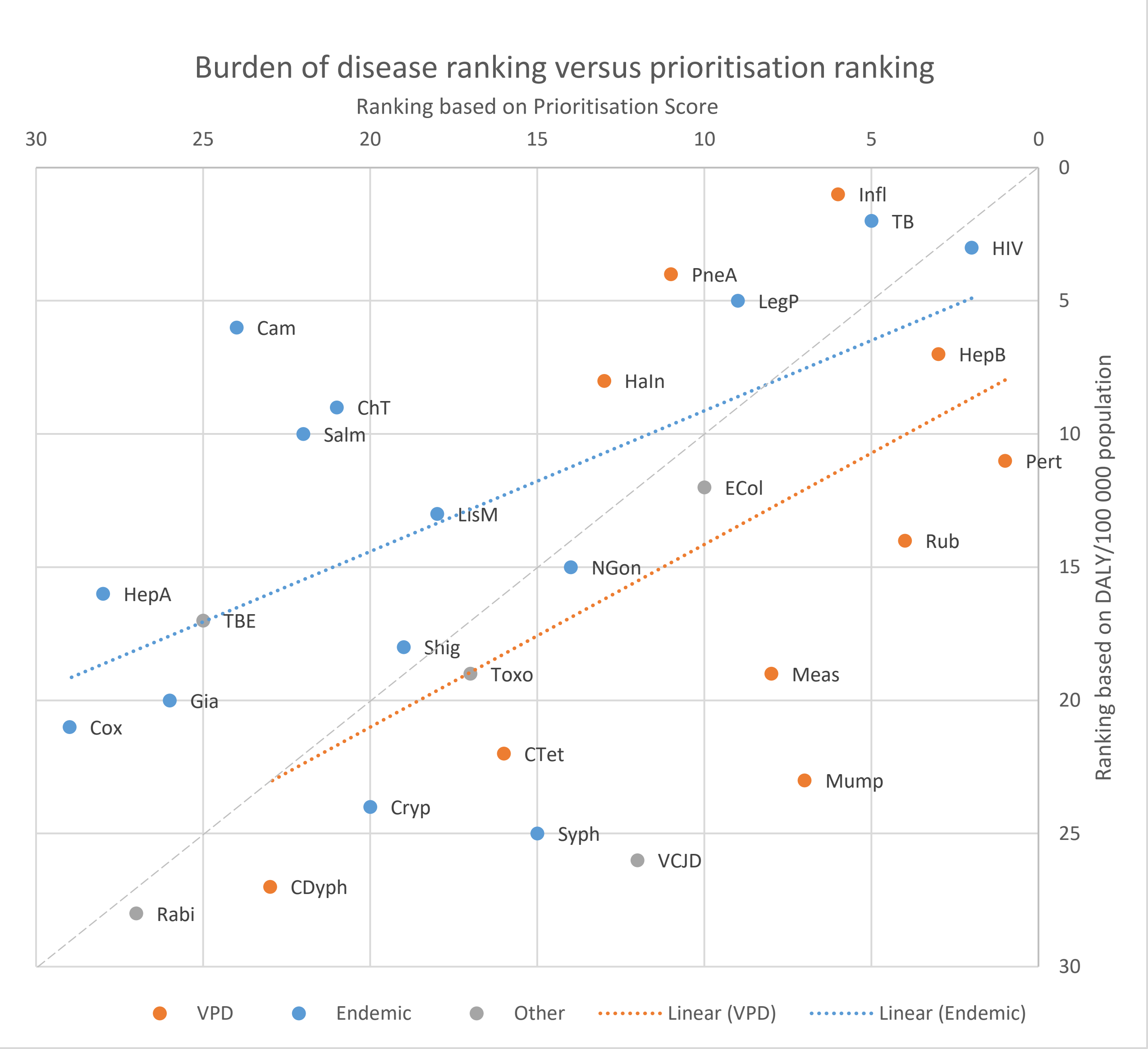

VPD=vaccine preventable diseases; Endemic=endemic diseases; Disease abbreviations are presented below Figure 5.

Cassini *et al*. Eurosurveillance 2018
https://www.eurosurveillance.org/content/10.2807/1560-7917.ES.2018.23.16.17-00454

Figure S10: Ranks of 89 diseases in the prioritisation study in Belgium 2018 compared to the ranks in the prioritisation study in Germany 2011 (Balabanova, 2011). Only endemic (n=34) and vaccine preventable diseases (n=14) are shown. Note that axes values are shown in reverse order, to emphasize that lower ranks represent higher priority. Our study ranked vaccine preventable diseases higher compared to the German study of 2011 (except for HPV and pneumococcal disease which were ranked higher in the German study; and hepatitis B and *Neisseria meningitides*, which were ranked almost equal in both studies). Disease abbreviations are presented below Figure 5.

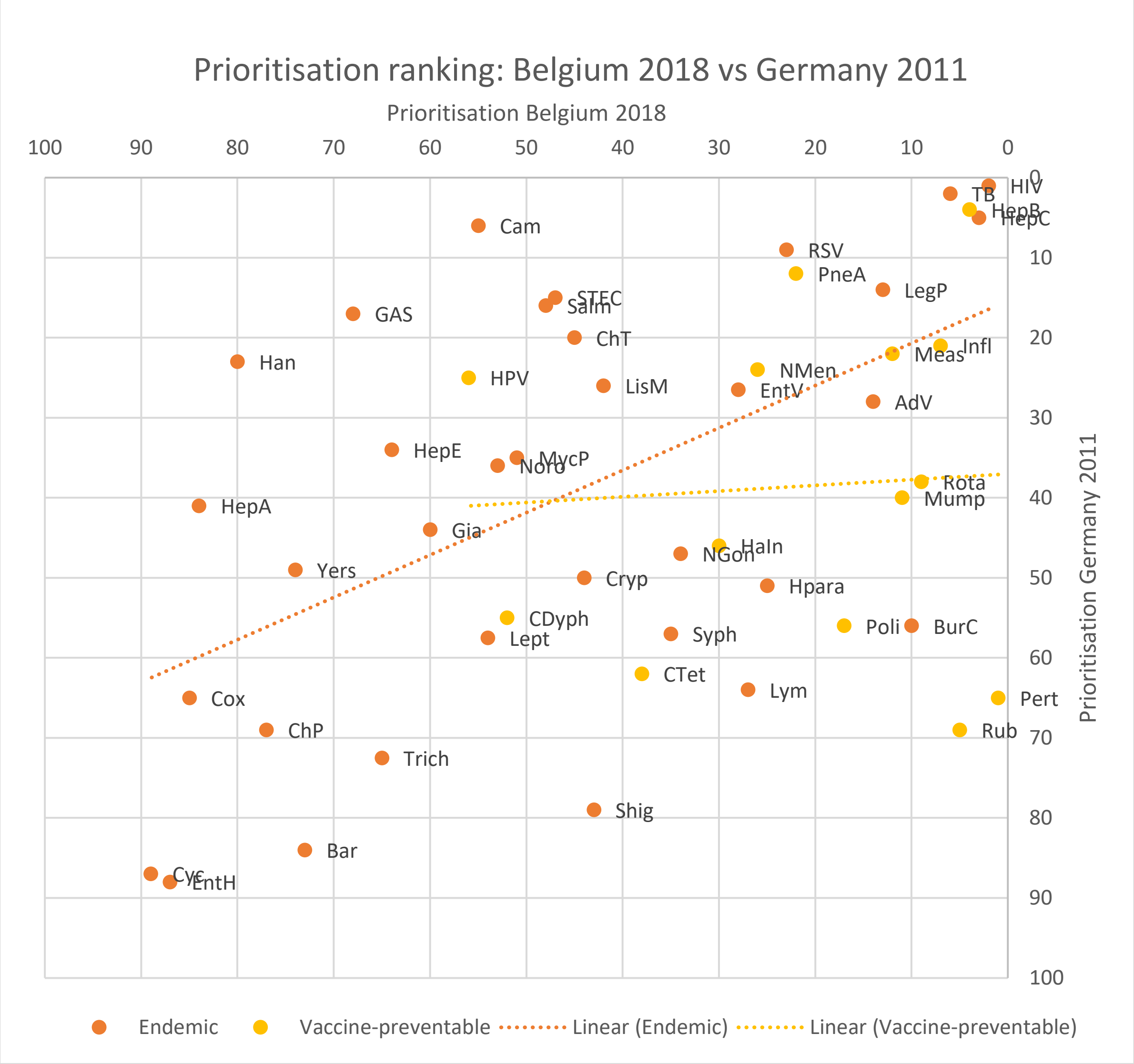

FULL DEFINTIONS OF THE CRITERIA:

INCIDENCE AND TREND

Incidence: Total number of symptomatic cases annually in Belgium. This is not always equal to the number of reported cases by the national surveillance systems. Correction factors for the estimated underdiagnoses and underreporting should be applied, in order to obtain the estimated total number of symptomatic cases. This includes both imported and autochthonous cases.

Trend: Trend of the incidence for each pathogen in Belgium over the period 2010-2016. This again concerns the trend of the estimated total number of symptomatic cases. This is not always equal to the observed trend in surveillance data (e.g. in case of increased testing for this pathogen over the years).

IMPACT ON THE PATIENT

Case-fatality ratio*: Percentage of lethal cases among all symptomatic cases annually in Belgium.

Severity*: Perceived severity of the pathogen in Belgium, i.e. distribution of the clinical presentation of all symptomatic cases. This represents the discomfort at individual level for the patient.

Chronicity and/or chronic sequelae*: Percentage of patients that experience chronic disease (>6 months) and/or have serious sequelae relative to all symptomatic cases in Belgium.

Assessed **for each particular pathogen in question, relative to all symptomatic cases provoked by that particular pathogen.*

IMPACT ON SOCIETY

Work and school absenteeism*: Absenteeism due to the total burden of disease caused by each pathogen (not per case, but for all cases) relative to the total absenteeism due to infectious illness in Belgium.

Excess costs*: Direct and indirect costs due to the total burden of disease caused by each pathogen (not per case, but for all cases) relative to the total excess costs due to infectious illness in Belgium.

Health care utilization*: Health care utilization (primary care and hospitalization) due to the total burden of disease caused by each pathogen (not per case, but for all cases) relative to the total health care utilization due to infectious illness in Belgium.

Public attention*: Risk perception among the general population, amount of media attention and ranking on the political agenda. For infections that did not occur during the reference period, this criteria can be considered as the public attention that the pathogen will attract in the scenario that one case will occur.

Assessed is **the total burden of one infectious disease (all cases, 1 disease) relative to the total burden of all infectious diseases in Belgium (all cases, all infectious diseases).*

IMPACT ON PUBLIC HEALTH

Spreading potential: Perceived spreading potential of the pathogen. Indicators for the spreading potential are the theoretical reproductive number of the pathogen (R0: the reproduction of infections in a completely homogeneous and susceptible population), the mode of transmission (transmission by aerosols or droplets usually indicates high spreading potential) and prevention possibilities.

Proportion of events requiring public health action: Percentage of events provoked by the pathogen that require urgent public health actions. Event is defined as the occurrence of disease that is unusual and/or exceeding base-line levels with respect to a particular time, place and circumstances. Public health actions are any kind of targeted actions aimed to identify the nature of the event and/or to apply control measures in response to the event.

SURVEILLANCE NEEDS

WHO objective for eradication or elimination: Some diseases are target of specific eradication or elimination programs by the WHO. These disease control programs require targeted surveillance and rapid response capabilities at the national level.

International obligations for surveillance: All diseases that are included in the WHO, ECDC and/or OIE surveillance programs. National surveillance data is reported to the international surveillance programs.

Existing multidrug resistance: The presence of drugs resistance (antibiotics, antivirals, ect) other than those inherent to the specific species. The definition of multidrug-resistance varies per pathogen (e.g. resistance to 3 different drug classes).

Vaccine included in NVP: All pathogens that are included in the national vaccination program (NVP) for the general population. This accounts for the lower incidence of vaccine-preventable diseases due to effective prevention programs and the need to maintain adequate surveillance capacities for these pathogens.

Risk for vaccine triggered strain replacement: Risk for increased incidence of formerly subdominant types or species after vaccination (vaccination can drive the emergence of formerly subdominant strains).

Congenital risk: Risk for mother-to-child transmission of an infectious disease AND serious complications for the child.

NRC/RefLab essential for diagnosis: All pathogens for which a national laboratory is essential for the first diagnosis of the patients AND the patients' treatment. This includes pathogens for which the national laboratory is essential for the diagnosis AND treatment of only a proportion of patients (e.g. difficult cases). This concerns the diagnosis at genus-level and does not include typing or antibiograms of pathogens.
