# Supplementary material for: Prioritisation for future surveillance, prevention and control of 98 communicable diseases in Belgium: a 2018 multi-criteria decision analysis study
Source: BMC Public Health. 2021 Jan 22;21:192. doi: 10.1186/s12889-020-09566-9 (PMC7820105; doi:10.1186/s12889-020-09566-9)
Supplement: Supplementary file 2 — Additional file 2:. “Survey for weights”. [file 12889_2020_9566_MOESM2_ESM.pdf]

Prioritization: assigning weights to criteria

Evidence-based prioritization of infectious diseases in Belgium

---

Context

Many pathogens present public health threats. For a relative comparison between pathogens, many aspects should be considered. The objective of this study is to **prioritize** multiple pathogens, according to their relative importance for **public health and surveillance**.

A **multi-criteria decision analysis** (MCDA) model allows an objective and evidence-based comparison between pathogens. The MCDA-approach uses a balanced set of (semi-) quantitative **criteria,** that together quantify the impact on public health per pathogen. The criteria are not all equally important for the overall risk, therefore, the individual criteria should be weighted according to their relative contribution to the overall risk.

In this survey (Survey I), we invite a panel of experts to assign a weight to each criterion, according to their expert opinion. This survey is part of the prioritisation process, described by the ECDC tool for prioritization:

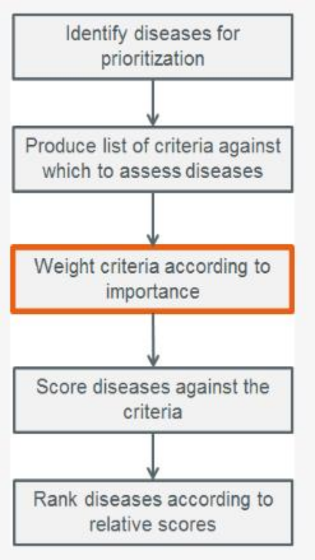

Sciensano · Rue Juliette Wytsman 14 · 1050 Bruxelles · Belgique
T + 32 2 642 51 11 · F + 32 2 642 50 01 · info@sciensano.be · www.sciensano.be

toute une vie en bonne santé

sciensano

The next step will be to score each pathogen against each criteria (Survey II). The **overall weighted scores** per pathogen will be used to **rank the pathogens**. The results of this study will support priority setting within the public health domain and the development of surveillance activities. Please note, survey I (this one) and survey II (next one) are completely independent from each other, and participation in this first survey is completely without any further obligations.

For more detailed information, an example of a similar prioritization exercise and the current study protocol are available in the links below.

Practical information

This survey includes **18 criteria**, that are organized in the following **5 criteria groups**:

- Surveillance needs
- Impact on society
- Impact on public health
- Impact on the patient
- Incidence & trend

First, you are kindly asked to assign **a value ranging from 1 to 10 to each criterion,** considering its importance **within the criterion group.** The assigned value should reflect the relative importance of the criterion for public health and surveillance. The value of 1 reflects the lowest and 10 the highest level of importance.

Secondly, you are kindly asked to assign **a value ranging from 1 to 10 to each criterion group.** The assigned value should reflect the relative importance of the criterion group for public health and surveillance. The value of 1 reflects the lowest and 10 the highest level of importance.

The final weights of the criteria will be determined as the individual weight of the criterion multiplied by the weight of its criterion group (**hierarchical weighting**). More than one criterion can be assigned the same weight.

This survey will take **approximately 15-20 minutes**.

References

- ECDC guidelines for prioritisation.
- An example of a similar prioritization exercise.
- The summary of the current study protocol.

NOTE: full definitions of all criteria are provided on the last page of this survey.

sciensano

Section A: BACKGROUND

What is your profession and/or your professional background?
Please select the answer that fits best.

- ○ Clinician
- ○ Microbiological laboratory expert
- ○ Public health specialist/ Epidemiologist
- ○ Other, ____

Which discipline is your main field of action?

- ○ Human
- ○ Animal
- ○ Food/ feed/ environmental

What is your native language?

- ○ Dutch
- ○ French
- ○ Other, ____

What is your gender?

- ○ Female
- ○ Male

sciensano

Section B: WEIGHTING

Please assign a weight between 1 and 10 to each of the following criteria within each criterion group (1=not important; 10=highly important).

The assigned weight should reflect the relative importance of these criteria for the overall score, according to your opinion. The overall score will be a combination of this survey and the next survey, and represents the impact of the pathogen on public health and surveillance.

NOTE: full definitions of the criteria are provided at the last page.

NOTE: multiple criteria can be assigned the same weight.

Please indicate your choice by moving the slider on the scale-bar:

1 2 3 4 5 6 7 8 9 10

B1: Surveillance needs

WHO objective for eradication or elimination:

1 2 3 4 5 6 7 8 9 10

International obligations for surveillance:

1 2 3 4 5 6 7 8 9 10

National reference laboratory (NRC/RefLab) essential for diagnosis:

1 2 3 4 5 6 7 8 9 10

Existing multi-drug resistance:

1 2 3 4 5 6 7 8 9 10

Vaccine included in the national vaccination program (NVP):

1 2 3 4 5 6 7 8 9 10

Risk for vaccine-triggered strain replacement:

1 2 3 4 5 6 7 8 9 10

Congenital risks:

1 2 3 4 5 6 7 8 9 10

toute une vie en bonne santé

B2: Impact on society

WHO objective for eradication or elimination:

1 2 3 4 5 6 7 8 9 10

International obligations for surveillance:

1 2 3 4 5 6 7 8 9 10

National reference laboratory (NRC/RefLab) essential for diagnosis:

1 2 3 4 5 6 7 8 9 10

Existing multi-drug resistance:

1 2 3 4 5 6 7 8 9 10

B3: Impact on public health

WHO objective for eradication or elimination:

1 2 3 4 5 6 7 8 9 10

International obligations for surveillance:

1 2 3 4 5 6 7 8 9 10

B4: Impact on the patient

WHO objective for eradication or elimination:

1 2 3 4 5 6 7 8 9 10

International obligations for surveillance:

1 2 3 4 5 6 7 8 9 10

National reference laboratory (NRC/RefLab) essential for diagnosis:

1 2 3 4 5 6 7 8 9 10

B5: Incidence and trend

WHO objective for eradication or elimination:

1 2 3 4 5 6 7 8 9 10

International obligations for surveillance:

1 2 3 4 5 6 7 8 9 10

Section C: GROUP WEIGHTS

Please assign a weight between 1 and 10 to each of the following criteria groups reflecting the relative importance of the group within the overall score (1 = not important; 10 = highly important).

Surveillance needs:

1 2 3 4 5 6 7 8 9 10

Impact on society:

1 2 3 4 5 6 7 8 9 10

Impact on public health:

1 2 3 4 5 6 7 8 9 10

Impact on the patient:

1 2 3 4 5 6 7 8 9 10

Incidence and trend:

1 2 3 4 5 6 7 8 9 10

Section D: FINAL QUESTION

Please, complete the previous pages first. Once completed, please, do not look back to previous pages.

Rank the following individual criteria, according to their relative importance for public health (highest importance=1; middle importance=2; lowest importance=3):

- ☐ Incidence
- ☐ Case-fatality ratio
- ☐ Excess costs

THANK YOU!

Thank you gratefully for your kind participation! Please, do not forget to SUBMIT!

If you would like to receive an invitation for the next survey (scoring of the pathogens against the criteria) and/or would like to get informed about the final results, feel free to leave your email address below:

Email: ____

If you have any comments/feedback/suggestions related to this survey or the ranking of infectious diseases in general, please feel free to write them down below:

CRITERIA DEFINITIONS (FYI)

Surveillance needs:

WHO objective for eradication or elimination: Some diseases are target of specific eradication or elimination programs by the WHO. These disease control programs require targeted surveillance and rapid response capabilities at the national level.

International obligations for surveillance: All diseases that are included in the WHO, ECDC and/or OIE surveillance programs. National surveillance data is reported to the international surveillance programs.

Existing multidrug resistance: The presence of drugs resistance (antibiotics, antivirals, ect) other than those inherent to the specific species. The definition of multidrugs resistance varies per pathogen (e.g. resistance to 3 different drug classes).

Vaccine included in NVP: All pathogens that are included in the national vaccination program (NVP) for the general population. This accounts for the lower incidence of vaccine-preventable diseases due to effective prevention programs and the need to maintain adequate surveillance capacities for these pathogens.

Risk for vaccine triggered strain replacement: Risk for increased incidence of formerly subdominant types or species after vaccination (vaccination can drive the emergence of formerly subdominant strains).

Congenital risk: Risk for mother-to-child transmission of an infectious disease AND serious complications for the child.

NRC/RefLab essential for diagnosis: All pathogens for which a national laboratory is essential for the first diagnosis of the patients AND the patients' treatment. This includes pathogens for which the national laboratory is essential for the diagnosis AND treatment of only a proportion of patients (e.g. difficult cases). This concerns the diagnosis at genus-level and does not include typing or antibiograms of pathogens.

Impact on society:

Work and school absenteeism*: Absenteeism due to the total burden of disease caused by each pathogen (not per case, but for all cases) relative to the total absenteeism due to infectious illness in Belgium.

Excess costs*: Direct and indirect costs due to the total burden of disease caused by each pathogen (not per case, but for all cases) relative to the total excess costs due to infectious illness in Belgium.

Health care utilization*: Health care utilization (primary care and hospitalization) due to the total burden of disease caused by each pathogen (not per case, but for all cases) relative to the total health care utilization due to infectious illness in Belgium.

Public attention*: Risk perception among the general population, amount of media attention and ranking on the political agenda. For infections that did not occur during the reference period, this criteria can be considered as the public attention that the pathogen will attract in the scenario that one case will occur.

**Assessed is the total burden of one infectious disease (all cases, 1 disease) relative to the total burden of all infectious diseases in Belgium (all cases, all infectious diseases).*

Impact on public health:

Spreading potential: Perceived spreading potential of the pathogen. Indicators for the spreading potential are the theoretical reproductive number of the pathogen (R0: the reproduction of infections in a completely homogeneous and susceptible population), the mode of transmission (transmission by aerosols or droplets usually indicates high spreading potential) and prevention possibilities.

Proportion of events requiring public health action: Percentage of events provoked by the pathogen that require urgent public health actions. Event is defined as the occurrence of disease that is unusual and/or exceeding base-line levels with respect to a particular time, place and circumstances. Public health actions are any kind of targeted actions aimed to identify the nature of the event and/or to apply control measures in response to the event.

Impact on the patient:

Case-fatality ratio*: Percentage of lethal cases among all symptomatic cases annually in Belgium.

Severity*: Perceived severity of the pathogen in Belgium, i.e. distribution of the clinical presentation of all symptomatic cases. This represents the discomfort at individual level for the patient.

Chronicity and/or chronic sequelae*: Percentage of patients that experience chronic disease (>6 months) and/or have serious sequelae relative to all symptomatic cases in Belgium.

**Assessed for each particular pathogen in question, relative to all symptomatic cases provoked by that particular pathogen.*

Incidence and trend:

Incidence: Total number of symptomatic cases annually in Belgium. This is not always equal to the number of reported cases by the national surveillance systems. Correction factors for the estimated underdiagnosis and underreporting should be applied, in order to obtain the estimated total number of symptomatic cases. This includes both imported and authochtoneous cases.

Trend: Trend of the incidence for each pathogen in Belgium over the period 2010-2016. This again concerns the trend of the estimated total number of symptomatic cases. This is not always equal to the observed trend in surveillance data (e.g. in case of increased testing for this pathogen over the years).
