# Supplementary material for: Prioritisation for future surveillance, prevention and control of 98 communicable diseases in Belgium: a 2018 multi-criteria decision analysis study
Source: BMC Public Health. 2021 Jan 22;21:192. doi: 10.1186/s12889-020-09566-9 (PMC7820105; doi:10.1186/s12889-020-09566-9)
Supplement: Supplementary file 3 — Additional file 3:. “Survey for scores”. [file 12889_2020_9566_MOESM3_ESM.pdf]

Prioritization:

Evidence-based prioritization of infectious diseases in Belgium

Context

Many pathogens present public health threats. For a relative comparison between pathogens, many aspects should be considered. The objective of this study is to prioritize multiple pathogens, according to their relative impact on public health and relevance for surveillance.

A multi-criteria decision analysis (MCDA) model allows an objective and evidence-based comparison between pathogens. This approach uses a balanced set of (semi-)quantitative criteria, that together quantify the impact on public health per pathogen. The criteria are not all equally important for the overall risk, therefore, the individual criteria are weighted according to their relative contribution to the overall risk.

In a previous survey (Survey I), we invited a panel of experts to assign a weight to each criterion, according to their expert opinion. This survey (Survey II) focusses on scoring each pathogen against each criteria, again by a multidisciplinary panel of experts. The overall weighted scores per pathogen will be used to rank the pathogens. The results of this study will support priority setting within the public health domain and the development of surveillance activities.

The estimated time to complete this survey, depends on the number of pathogens you like to include in your answers. We encourage you to include as many pathogens as possible, especially for the questions fully based on expert opinions. The estimated time to complete this survey is between 25-50 minutes. We realize that your time is very precious and therefore we provided default answers for some questions. We hope you allow yourselves this time investment, because the validation of the values per pathogen is essential for the ranking of pathogens.

This survey is part of the prioritisation process, described by the ECDC tool for the prioritization of infectious disease threats :

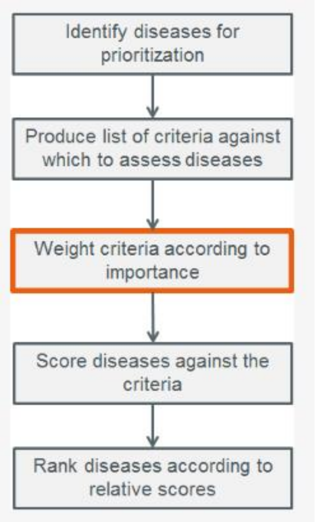

Sciensano · Rue Juliette Wytsman 14 · 1050 Bruxelles · Belgique
T + 32 2 642 51 11 · F + 32 2 642 50 01 · info@sciensano.be · www.sciensano.be

An example of a similar prioritization exercise can be found here.
More details about the current study can be found in the study protocol.

Practical information

This survey includes questions and information about **98 pathogens**, that are organized in the following **groups**:

- Vaccine preventable pathogens
- Endemic/autochtonous acquired pathogens under national surveillance
- Imported and/or very rare pathogens under national surveillance
- Limited surveillance and/or congenital surveillance
- Invasive infections, community- or hospital-acquired (including invasive mycosis and AMR surveillance)

All questions should be answered according to the context of **Belgium and the period 2010-2016**.
We provide a link to **'help data'** that can be consulted in order to base your answer on available data and existing evidence. You can skip certain disease groups or pathogens, but we encourage you to include as many pathogens as possible in your answers, especially for the questions fully based on expert opinions.

Scoring

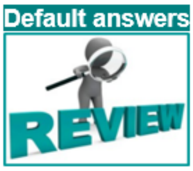

For the quantitative (objective) criteria, **default answers** are defined based on existing evidence and validated by internal experts. We kindly ask you to **review** these answers and select any alternative answer when you do not fully agree with the interpretation of the internal experts (the default answer).

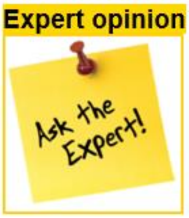

For some criteria insufficient data is available, and therefore we rely on your **expert opinion** ('help data' can be consulted).

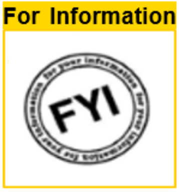

A concluding set of variables is included for your information, but you are welcome to comment on the provided tables.

Question outline

1. Background of the participant
2. The following criteria are included for the MCDA-analysis:
   - INCIDENCE & TREND
     - **Incidence** (default answers provided)
     - **Trend** (default answers provided)
   - IMPACT ON THE PATIENT (individual impact, burden per case)
     - **Case-fatality** (default answers provided)
     - **Severity** (default answers provided)
     - **Chronicity** (default answers provided)
   - IMPACT ON SOCIETY (society impact, total burden of all cases)
     - **School and work absenteeism** (full expert opinion)
     - **Health care utilization** (full expert opinion)
     - **Excess costs** (full expert opinion)
   - IMPACT ON PUBLIC HEALTH:
     - **Spreading potential (contagiousness)** (default answers provided)
     - **Events requiring public health action** (full expert opinion)
     - **Surveillance needs** (for your information)
3. Additional open question about:
   - Pathogens for which surveillance should be improved significantly
   - Pathogens with a high probability that the impact increases in the next 10 years
   - Additional pathogens with considerable impact on public health that were not covered by this survey

---

Navigation

- You can navigate through the pages by 'next' and 'previous' on the bottom of each page.
- In addition, you can navigate by the 'question index' on the top right of each page.
- You can save your answers intermediately to resume later by use of the button 'resume later'.
- Questions with a red asterisk (*) are mandatory and need completion before you can proceed.

YOUR PARTICIPATION WILL BE MUCH APPRECIATED!

Section A: BACKGROUND

What is your profession and/or your professional background?
Please select the answer that fits best.

- ○ Clinician, specialized in infectious diseases
- ○ Clinician, specialized in pediatric diseases
- ○ Clinician (other)
- ○ Microbiological laboratory expert
- ○ Public health specialist/ Epidemiologist
- ○ Other, ____

Which discipline is your main field of action?

- ○ Human
- ○ Animal
- ○ Food/ feed/ environmental

What is your native language?

- ☐ Dutch
- ☐ French
- ☐ Other, ____

What is your gender?

- ○ Female
- ○ Male

Section B:

INCIDENCE AND TREND
B1: INCIDENCE

Default answers

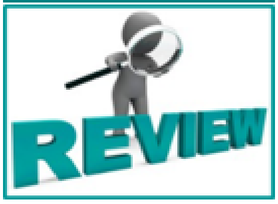

QUESTION B1 - INCIDENCE:

What is the estimated average total number of symptomatic cases annually in Belgium for each pathogen considering the 2010-2016 period, in your opinion?

FULL DEFINITION: The incidence is estimated as the average total number of symptomatic cases annually in Belgium. This is not always equal to the number of reported cases by the national surveillance systems. Correction factors for the estimated underdiagnoses and underreporting should be applied, in order to obtain the estimated total number of symptomatic cases. This includes both imported and autochthonous cases.

LEVELS (i.e. answer categories):

- Rare (<10 symptomatic cases annually)
- Low (10-99 symptomatic cases annually)
- Moderate (100-999 symptomatic cases annually)
- High (>=1000 symptomatic cases annually)

Important note:

- We ask to consider the average total number of symptomatic cases, i.e. the number of reported cases multiplied by a correction factor for under-diagnosis and under-reporting.
- To account for outbreak prone pathogens (e.g. measles) with highly varying incidence levels per year, we ask to estimate the average yearly number of symptomatic cases over the 2010-2016 period.

The following default answers are proposed.
Available 'help data' for the incidence (number of reported cases, lab-confirmed cases and estimate for total symptomatic cases) can be consulted here.

INCIDENCE - VACCINE PREVENTABLE PATHOGENS (NATIONAL VACCINATION PROGRAM):

Rare<br>0-10 cases per year	Low<br>10-99 cases per year	Moderate<br>100-999 cases per year	High<br>>1000 cases per year
Clostridium tetani		Haemophilus influenza B	Mumps
Corynebacterium (C. diphtheriae, C. ulcerans, C. pseudotuberculosis)		HPV	Bordetella pertussis
Poliovirus		Invasive pneumo in children (≤15 y)	Hepatitis B virus
Rubella (congenital)		Measles	Influenza
		Meningococcus (*Neisseria meningitidis*)	Invasive pneumo in adults (16+ y)
			Rotavirus

sciensano

INCIDENCE - ENDEMIC/AUTOCHTHONOUS PATHOGENS:

Rare<br>0-10 cases per year	Low<br>10-99 cases per year	Moderate<br>100-999 cases per year	High<br>>1000 cases per year
	Anaplasma	Burkholderia cepacia complex	Adenovirus
	Chlamydia psittaci (parrot fever)	Hantavirus	Bartonella (B. henselae)
	Coxiella burnetii (Q fever)	HAV	Borrelia burgdorferi senu lato (Lyme disease)
	Cyclospora	HEV	Campylobacter
	Echinococcus multilocularis	Legionella pneumophila	Chlamydia trachomatis +LVG
	Entamoeba histolytica	Listeria monocytogenes	Cryptosporidium sp.
	Leptospira	Mycobacterium tuberculosis	Giardia
		Shiga-toxin producing E. coli	Hepatitis C virus
		Streptococcus pyogenes (SGA/GAS) (invasive)	HIV
			Humaan metapneumovirus (HMPV)
			Human parainfluenzavirus
			Mycoplasma pneumoniae
			Neisseria gonorrhoeae
			Non-polio enteroviruses and parechoviruses
			Noroviruses
			Respiratoir syncitieel virus (RSV)
			Salmonella (non-typhoid)
			Shigella
			Treponema pallidum
			Yersinia enterocolitica and Yersinia pseudotuberculosis

sciensano

INCIDENCE - IMPORTED AND/OR VERY RARE PATHOGENS:

Rare<br>0-10 cases per year	Low<br>10-99 cases per year	Moderate<br>100-999 cases per year	High<br>>1000 cases per year
Bacillus anthracis	Chikungunya (arbovirus)	Dengue (arbovirus)	
Brucella.spp	Leishmania	Plasmodium (malaria)	
Burkholderia mallei/pseudo mallei	Rickettsia	Zika (arbovirus)	
Clostridium botulinum	Salmonella Typhoid		
Coronavirus including MERS, SARS			
Ebola			
Francisella tularensis (tularaemia)			
Mycobacterium leprae			
Rabies virus			
Smallpox			
Tick-borne encephalitis (arbovirus)			
Trichinosis / trichinellosis			
Tropheryma whipplei			
Trypanosoma cruzi (Chagas disease)			
VCJD			
Vibrio cholerae and Vibrio parahaemolyticus			
West Nile virus (arbovirus)			
Yellow fever virus (arbovirus)			

INCIDENCE - LIMITED SURVEILLANCE AND/OR CONGENITAL SURVEILLANCE ONLY:

Rare<br>0-10 cases per year	Low<br>10-99 cases per year	Moderate<br>100-999 cases per year	High<br>>1000 cases per year
Parvovirus B19 (congenital)	Babesia	Bacillus cereus	Chlamydia pneumoniae
Toxoplasmose (congenital)	Streptococcus agalactiae (GBS) (invasive and/or congenital)	CMV (congenital)	Helicobacter pylori
		Herpes simplex (in CSF)	Herpes zoster varicella (zona, varicella) (in CSF)
		Pasteurella	
		Scabies	

INCIDENCE - HOSPITAL RELATED INFECTIONS (INCL. INVASIVE MYCOSIS & AMR):

Rare<br>0-10 cases per year	Low<br>10-99 cases per year	Moderate<br>100-999 cases per year	High<br>>1000 cases per year
	Cryptococcus (invasive)	Aspergillus (invasive)	Candida spp (invasive)
	Acinetobacter (invasive) (including MDR)	Enterococci (including VRE) (E. faecalis and E. faecium) (invasive)	Clostridium difficile
		Klebsiella (invasive)	E. coli (non STEC/EHEC) invasive
		Pseudomonas (including MDR)	Staphylococcus aureus (including MRSA) (invasive)

QUESTION B1 - INCIDENCE:
Do you agree with the default answers in the tables above (based on available data), or do some pathogens belong to another category in your opinion?

We rely on your expert opinion because:

- For some pathogens limited data was available in general (especially for the group of pathogens 'limited surveillance').
- For other pathogens surveillance data is available, but data about the amount of underreporting (coverage of the surveillance) and under ascertainment (patients that do not seek health care and/or a lab-sample is not taken) might be limited.
- Ideas about the real incidence of symptomatic cases might diverge among experts.

Please select the appropriate response for each line:

	I agree on the default answers for all pathogens in this group	I would like to change the answer for 1 or more pathogen(s) in this group	I have no opinion for this group
**Vaccine preventable pathogens (within the national vaccination program)**	○	○	○
**Endemic/autochtonous pathogens under surveillance**	○	○	○
**Imported and/or very rare pathogens under surveillance**	○	○	○
**Limited surveillance and/or congenital surveillance only**	○	○	○
**Hospital related infections (including invasive mycosis and AMR surveillance)**	○	○	○

We would like to know whether you agree with the default answers, whether you want to change the default answers of certain pathogen(s) within certain pathogen group(s), or whether you do not have an opinion. Please, indicate one of these three options for each group of pathogens.

If you indicate that you want to change one or more pathogens within a certain pathogen group, please indicate the corrections in the question(s) that appear upon selection of the pathogen group(s). Again, you have the option for each pathogen to:

- confirm the default answers (leave the default answer unchanged)
- correct the default answer (change the default answer to another category)
- or indicate that you do not know.

[FOR REASONS OF CLARITY, THE FULL TABLE OF 98 DISEASE IS NOT SHOWN IN THIS PAPER VERSION OF THE SURVEY. THE TABLE LOOKS LIKE THE FOLLOWING (pre-filled according to default answer tables above):]

Disease / pathogen	Rare 0-10 cases per year	Low 10-99 cases per year	Moderate 100-999 cases per year	High >1000 cases per year	Do not know
**Disease 1**	○	○	○	○	○
**Disease 2**	○	○	○	○	○
**Disease …**	○	○	○	○	○

Section B - continuation

INCIDENCE AND TREND
B2: TREND

Default answers

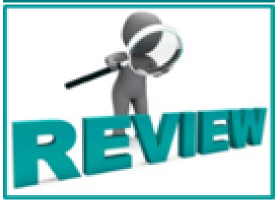

QUESTION B2 - TREND:

What is the trend of the incidence for each pathogen in Belgium considering the 2010-2016 period, in your opinion?

FULL DEFINITION: Trend of the incidence for each pathogen in Belgium over the period 2010-2016. This again concerns the trend of the estimated total number of symptomatic cases. This is not always equal to the observed trend in surveillance data (e.g. in case of increased testing for this pathogen over the years).

LEVELS (i.e. answer categories):

- Decline
- No apparent decline or increase
- Increase

Important note:

- The option 'no apparent decline or increase' contains both pathogens with a true stable evolution of the incidence over time, as pathogens for which not enough information is available to estimate the trend (i.e. unknown).
- The identification of a trend highly depends on the time window of interest. In order to keep the cross-pathogen comparison valid, please consider the complete reference period of 2010-2016.
  - When a pathogen declined in incidence during 2011-2013 but highly increased during 2015-2016, a linear trend will not be identified over the total period (2010-2016), although the incidence of the pathogen recently increased (which might even prompt robust public health actions).
  - The incidence of some pathogens shows a highly year-by-year variation, therefore, a 6-year trend might be a better indication for a stable increase or decline than a 1- or 2-year trend. For pathogens that are highly outbreak sensitive (e.g. mumps, measles, HAV), linear trends are often not detected, but this does not mean that no recent outbreaks took place.
- We are interested in the trend of the estimated number of symptomatic cases, not in the increase or decrease of the number of reported cases. Therefore, attributable factors (e.g. increase in testing) should be taken into account.

The following default answers are proposed.
Available 'help data' for the trend of the incidence can be consulted here.

TREND - VACCINE PREVENTABLE PATHOGENS (NATIONAL VACCINATION PROGRAM):

Decline	No apparent decline or increase	Increase
Invasive pneumo in adults (16+)	[OTHERS]	Bordetella pertussis
Invasive pneumo in children (≤15)		

TREND - ENDEMIC/AUTOCHTHONOUS PATHOGENS:

Decline	No apparent decline or increase	Increase
Entamoeba histolytica	[OTHERS]	Adenovirus
		Burkholderia cepacia complex
		Chlamydia trachomatis +LVG
		Cryptosporidium sp.
		HEV
		Human parainfluenzavirus
		Legionella pneumophila
		Leptospira
		Neisseria gonorrhoeae
		Non-polio enteroviruses and parechoviruses
		Treponema pallidum

TREND - IMPORTED AND/OR VERY RARE PATHOGENS:

Decline	No apparent decline or increase	Increase
x	OTHERS	Zika (arbovirus)
		Francisella tularensis (tularaemia)

TREND - LIMITED SURVEILLANCE AND/OR CONGENITAL SURVEILLANCE ONLY:

Decline	No apparent decline or increase	Increase
x	OTHERS	Scabies

TREND - HOSPITAL RELATED INFECTIONS (INCL. INVASIVE MYCOSIS & AMR):

Decline	No apparent decline or increase	Increase
x	OTHERS	Klebsiella (invasive)

QUESTION B2 - TREND:
Do you agree with the default answers in the tables above (based on available data), or do some pathogens belong to another category in your opinion?

We rely on your expert opinion because:

- For some pathogens limited data was available in general (especially for the group of pathogens 'limited surveillance').
- The attributing factors are subject of interpretation, and therefore expert opinions might diverge among experts.

Please select the appropriate response for each line:

	I agree on the default answers for all pathogens in this group	I would like to change the answer for 1 or more pathogen(s) in this group	I have no opinion for this group
**Vaccine preventable pathogens (within the national vaccination program)**	○	○	○
**Endemic/autochtonous pathogens under surveillance**	○	○	○
**Imported and/or very rare pathogens under surveillance**	○	○	○
**Limited surveillance and/or congenital surveillance only**	○	○	○
**Hospital related infections (including invasive mycosis and AMR surveillance)**	○	○	○

We would like to know whether:

- you agree with the default answers;
- you like to change the default answers of certain pathogen(s) within certain pathogen group(s);
- you do not have an opinion.

Please, indicate one of these three options for each group of pathogens.

If you indicate that you want to change one or more pathogens within a certain pathogen group, please indicate the corrections in the question(s) that appear upon selection of the pathogen group(s). Again, you have the option for each pathogen to:

- confirm the default answers (leave the default answer unchanged)
- correct the default answer (change the default answer to another category)
- or indicate that you do not know.

[FOR REASONS OF CLARITY, THE FULL TABLE OF 98 DISEASE IS NOT SHOWN IN THIS PAPER VERSION OF THE SURVEY. THE TABLE LOOKS LIKE THE FOLLOWING (pre-filled according to default answer tables above):]

Disease / pathogen	Decline	No apparent decline or increase	Increase	Do not know
**Disease 1**	○	○	○	○
**Disease 2**	○	○	○	○
**Disease …**	○	○	○	○

Section B - continuation

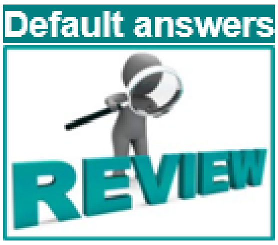

IMPACT ON PATIENT
B3: CASE-FATALITY-RATIO

QUESTION B3 – CASE-FATALITY-RATIO:

What is the case fatality ratio in Belgium for each pathogen relative to all symptomatic cases of this pathogen considering the 2010-2016 period, in your opinion?

FULL DEFINITION: Case-fatality ratio is defined as the percentage of lethal cases among all symptomatic cases annually in Belgium, considering the period 2010-2016.

LEVELS (i.e. answer categories):

- Rare (<0.01%)
- Low (0.01-1%)
- Moderate (1-10%)
- High (<10%)

Important note:

- For chronic diseases, the 'number of deaths directly related to the infection among cases in care' can be multiplied by 'the average time the current patients live with the diagnosis'. Alternatively, a rough estimate can be obtained by dividing the 'annual number of deaths directly related to the infection' by the 'annual number of new diagnoses'.

The following default answers are proposed.
Available 'help data' for the case-fatality-ratio can be consulted here.

CFR - VACCINE PREVENTABLE PATHOGENS (NATIONAL VACCINATION PROGRAM):

Rare (<0.01%)	Low (<1%)	Moderate (1-10%)	High (>10%)
Mumps	HPV	Corynebacterium diphtheriae, Corynebacterium ulcerans, Corynebacterium pseudotuberculosis	Clostridium tetani
Bordetella pertussis	Measles	Hepatitis B virus	Haemophilus influenza B
Influenza	Rubella (congenital)	Invasive pneumo in adults (16+ y)	Invasive pneumo in children (≤15 y)
Rotavirus		Poliovirus	Meningococcus (Neisseria meningitidis)

CFR - ENDEMIC/AUTOCHTHONOUS PATHOGENS:

Rare (<0.01%)	Low (<1%)	Moderate (1-10%)	High (>10%)
Adenovirus	Anaplasma	Hepatitis C virus	Burkholderia cepacia complex
Bartonella (B. henselae)	Chlamydia psittaci (parrot fever)	Leptospira	Legionella pneumophila
Borrelia burgdorferi senu lato (Lyme disease)	Coxiella burnetii (Q fever)	Mycobacterium tuberculosis	Listeria monocytogenes
Campylobacter	Echinococcus multilocularis	Shiga-toxin producing E. coli	Tropheryma whipplei
Chlamydia trachomatis +LVG	Hantavirus	Streptococcus pyogenes (SGA/GAS) (invasive)	
Cryptosporidium sp.	HAV	HIV	
Cyclospora	Neisseria gonorrhoeae		
Entamoeba histolytica			
Giardia			
HEV			
Human metapneumovirus (HMPV)			
Human parainfluenzavirus			
Mycoplasma pneumoniae			
Non-polio enteroviruses and parechoviruses			
Noroviruses			
Respiratoir syncitieel virus (RSV)			
Salmonella (non-typhoid)			
Shigella			
Treponema pallidum			
Yersinia enterocolitica and Yersinia pseudotuberculosis			

CFR - IMPORTED AND/OR VERY RARE PATHOGENS:

Rare (<0.01%)	Low (<1%)	Moderate (1-10%)	High (>10%)
Francisella tularensis (tularaemia)	Brucella.spp	Bacillus anthracis	Burkholderia mallei/pseudo mallei
Mycobacterium leprae	Chikungunya (arbovirus)	Trypanosoma cruzi (Chagas disease)	Coronavirus including MERS, SARS
Trichinosis / trichinellosis	Clostridium botulinum		Ebola
Vibrio cholerae and Vibrio parahaemolyticus	Dengue (arbovirus)		Rabies virus
Zika (arbovirus)	Leishmania		Smallpox
	Plasmodium (malaria)		Tropheryma whipplei
	Rickettsia		VCJD
	Salmonella Typhoid		Yellow fever virus (arbovirus)
	Tick-borne encephalitis (arbovirus)		
	West Nile virus (arbovirus)		

CFR - LIMITED SURVEILLANCE AND/OR CONGENITAL SURVEILLANCE ONLY:

Rare (<0.01%)	Low (<1%)	Moderate (1-10%)	High (>10%)
Babesia	Pasteurella	CMV (congenital)	
Bacillus cereus		Streptococcus agalactiae (GBS) (invasive and/or congenital)	
Chlamydia pneumoniae			
Helicobacter pylori			
Herpes simplex (in CSF)			
Herpes zoster varicella (zona, varicella) (in CSF)			
Parvovirus B19 (congenital)			
Scabies			
Toxoplasmose (congenital)			

CFR - HOSPITAL RELATED INFECTIONS (INCL. INVASIVE MYCOSIS & AMR):

Rare (<0.01%)	Low (<1%)	Moderate (1-10%)	High (>10%)
	Acinetobacter (invasive) (including MDR)	Aspergillus (invasive)	Candida spp (invasive)
	E. coli (non STEC/EHEC) invasive	Clostridium difficile	Cryptococcus (invasive)
	Enterococci (including VRE) (E. faecalis and E. faecium) (invasive)		
	Klebsiella (invasive)		
	Pseudomonas (including MDR)		
	Staphylococcus aureus (including MRSA) (invasive)		

QUESTION B3 – CASE-FATALITY-RATIO: Do you agree with the default answers in the tables above (based on available data), or do some pathogens belong to another category in your opinion?

We rely on your expert opinion because:

- For some pathogens limited data was available in general (especially for the group of pathogens 'limited surveillance').
- For some pathogens limited data is available about the case fatality ratio in Belgium. When data from other countries and/or areas was used, experts might have diverging ideas about how this translates to the Belgium situation.

Please select the appropriate response for each line:

	I agree on the default answers for all pathogens in this group	I would like to change the answer for 1 or more pathogen(s) in this group	I have no opinion for this group
**Vaccine preventable pathogens (within the national vaccination program)**	○	○	○
**Endemic/autochtonous pathogens under surveillance**	○	○	○
**Imported and/or very rare pathogens under surveillance**	○	○	○
**Limited surveillance and/or congenital surveillance only**	○	○	○
**Hospital related infections (including invasive mycosis and AMR surveillance)**	○	○	○

We would like to know whether:

- you agree with the default answers;
- you like to change the default answers of certain pathogen(s) within certain pathogen group(s);
- you do not have an opinion.

Please, indicate one of these three options for each group of pathogens.

If you indicate that you want to change one or more pathogens within a certain pathogen group, please indicate the corrections in the question(s) that appear upon selection of the pathogen group(s). Again, you have the option for each pathogen to:

- confirm the default answers (leave the default answer unchanged)
- correct the default answer (change the default answer to another category)
- or indicate that you do not know.

[FOR REASONS OF CLARITY, THE FULL TABLE OF 98 DISEASE IS NOT SHOWN IN THIS PAPER VERSION OF THE SURVEY. THE TABLE LOOKS LIKE THE FOLLOWING (pre-filled according to default answer tables above):]

Disease / pathogen	Rare (<0.01%)	Low (<1%)	Moderate (1-10%)	High (>10%)	Do not know
**Disease 1**	○	○	○	○	○
**Disease 2**	○	○	○	○	○
**Disease …**	○	○	○	○	○

Section B - continuation

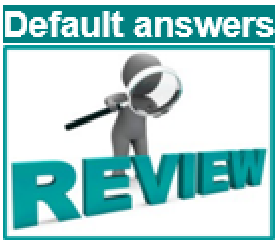

IMPACT ON PATIENT
B4: SEVERITY

QUESTION B4 – SEVERITY:

What is the perceived severity for each pathogen in Belgium considering the 2010-2016 time period, in your opinion?

FULL DEFINITION: Severity is defined as the distribution of the clinical presentation of all symptomatic patients. Perceived severity of the pathogen in Belgium, i.e. distribution of the clinical presentation of all symptomatic cases. This represents the discomfort at individual level for the patient. The severity of the clinical presentation is based on the necessary health-care assistance, according to the following definitions:

Clinical presentation	Description
Very mild clinical symptoms	Neglectable clinical symptoms or illness that is not noticeable
Mild clinical symptoms	Time off from work, some medical assistance or help at home
Moderate clinical symptoms	Urgent medical care and/or hospital admission
Severe clinical symptoms	Failure of major organ system(s) and/or long-term hospital admission

LEVELS (i.e. answer categories):
Most pathogens can provoke a range of clinical presentations, therefore we have defined the following levels (i.e. answer categories):

- Very mild - mild (moderate and severe cases less frequent)
- Mild - moderate (very mild and severe cases less frequent)
- Moderate - severe (very mild and mild cases less frequent)
- Mostly severe (very mild, mild and moderate cases less frequent)

Important note:

- The category should be chosen based on the distribution of the clinical presentation of all symptomatic patients. However, the distribution of clinical presentations provoked by a certain pathogen might not completely fit these definitions. For example, an infection might provoke very mild disease in most person and severe disease in a small but considerable fraction of patients. For this pathogen, category 2 or 3 might be chosen, although mild and moderate cases occur rarely.

The following default answers are proposed.
Available 'help data' for the case-fatality-ratio can be consulted here.

SEVERITY - VACCINE PREVENTABLE PATHOGENS (NATIONAL VACCINATION PROGRAM):

Very mild- Mild	Mild- Moderate	Moderate-Severe	Mostly Severe
Influenza	Mumps	Corynebacterium diphtheriae, Corynebacterium ulcerans, Corynebacterium pseudotuberculosis	Clostridium tetani
Rotavirus	Bordetella pertussis	Haemophilus influenza B	
	Hepatitis B virus	Invasive pneumo in adults (16+y)	
	HPV	Invasive pneumo in children (≤15y)	
	Measles	Meningococcus (Neisseria meningitidis)	
		Poliovirus	
		Rubella (congenital)	

SEVERITY - ENDEMIC/AUTOCHTHONOUS PATHOGENS:

Very mild- Mild	Mild- Moderate	Moderate-Severe	Mostly Severe
Adenovirus	Borrelia burgdorferi senu lato (Lyme disease)	Anaplasma	Burkholderia cepacia complex
Bartonella (B. henselae)	Chlamydia psittaci (parrot fever)	Coxiella burnetii (Q fever)	
Campylobacter	Coxiella burnetti (Q fever)	Echinococcus multilocularis	
Chlamydia trachomatis +LVG	Entamoeba histolytica	Hepatitis C virus	
Cryptosporidium sp.	Hantavirus	HIV	
Cyclospora	Listeria monocytogenes	Legionella pneumophila	
Giardia	Mycoplasma pneumoniae	Leptospira	
HAV	Respiratoir syncitieel virus (RSV)	Mycobacterium tuberculosis	
HEV	Shiga-toxin producing E. coli	Streptococcus pyogenes (SGA/GAS) (invasive)	
Humaan metapneumovirus (HMPV)	Shigella		
Human parainfluenzavirus			
Neisseria gonorrhoeae			
Non-polio enteroviruses and parechoviruses			
Noroviruses			
Salmonella (non-typhoid)			
Treponema pallidum			
Yersinia enterocolitica and Yersinia pseudotuberculosis			

SEVERITY - IMPORTED AND/OR VERY RARE PATHOGENS:

Very mild- Mild	Mild- Moderate	Moderate-Severe	Mostly Severe
Chikungunya (arbovirus)	Bacillus anthracis	Burkholderia mallei/pseudo mallei	Ebola
West Nile virus (arbovirus)	Brucella.spp	Coronavirus including MERS, SARS	Rabies virus
Zika (arbovirus)	Clostridium botulinum	Tropheryma whipplei	Smallpox
	Dengue (arbovirus)	Trypanosoma cruzi (Chagas disease)	VJCD
	Francisella tularensis (tularaemia)		Yellow fever virus (arbovirus)
	Leishmania		
	Mycobacterium leprae		
	Plasmodium (malaria)		
	Rickettsia		
	Salmonella Typhoid		
	Tick-borne encephalitis (arbovirus)		
	Trichinosis / trichinellosis		
	Vibrio cholerae and Vibrio parahaemolyticus		

SEVERITY - LIMITED SURVEILLANCE AND/OR CONGENITAL SURVEILLANCE ONLY:

Very mild- Mild	Mild- Moderate	Moderate-Severe	Mostly Severe
Babesia	CMV (congenital)	Streptococcus agalactiae (GBS) (invasive and/or congenital)	
Chlamydia pneumoniae	Bacillus cereus	Toxoplasmose (congenital)	
Helicobacter pylori	Herpes simplex (in CSF)		
Parvovirus B19 (congenital)	Herpes zoster varicella (zona, varicella) (in CSF)		
Scabies	Pasteurella		

SEVERITY - HOSPITAL RELATED INFECTIONS (INCL. INVASIVE MYCOSIS & AMR):

Very mild- Mild	Mild- Moderate	Moderate-Severe	Mostly Severe
		Acinetobacter (invasive) (including MDR)	Cryptococcus (invasive)
		Aspergillus (invasive)	
		Candida spp (invasive)	
		Clostridium difficile	
		E. coli (non STEC/EHEC) invasive	
		Enterococci (including VRE) (E. faecalis and E. faecium) (invasive)	
		Klebsiella (invasive)	
		Pseudomonas (including MDR)	
		Staphylococcus aureus (including MRSA) (invasive)	

QUESTION B4 – SEVERITY:
Do you agree with the default answers in the tables above (based on available data), or do some pathogens belong to another category in your opinion?

We rely on your expert opinion because:

- Severity is not a quantitative variable and conceived severity might diverge among experts.

Please select the appropriate response for each line:

	I agree on the default answers for all pathogens in this group	I would like to change the answer for 1 or more pathogen(s) in this group	I have no opinion for this group
**Vaccine preventable pathogens (within the national vaccination program)**	○	○	○
**Endemic/autochtonous pathogens under surveillance**	○	○	○
**Imported and/or very rare pathogens under surveillance**	○	○	○
**Limited surveillance and/or congenital surveillance only**	○	○	○
**Hospital related infections (including invasive mycosis and AMR surveillance)**	○	○	○

We would like to know whether:

- you agree with the default answers;
- you like to change the default answers of certain pathogen(s) within certain pathogen group(s);
- you do not have an opinion.

Please, indicate one of these three options for each group of pathogens.

If you indicate that you want to change one or more pathogens within a certain pathogen group, please indicate the corrections in the question(s) that appear upon selection of the pathogen group(s). Again, you have the option for each pathogen to:

- confirm the default answers (leave the default answer unchanged)
- correct the default answer (change the default answer to another category)
- or indicate that you do not know.

[FOR REASONS OF CLARITY, THE FULL TABLE OF 98 DISEASE IS NOT SHOWN IN THIS PAPER VERSION OF THE SURVEY. THE TABLE LOOKS LIKE THE FOLLOWING (pre-filled according to default answer tables above):]

Disease / pathogen	Very mild- Mild	Mild- Moderate	Moderate-Severe	Mostly Severe	Do not know
**Disease 1**	○	○	○	○	○
**Disease 2**	○	○	○	○	○
**Disease …**	○	○	○	○	○

Section B - continuation

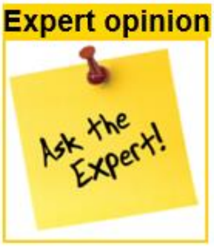

IMPACT ON SOCIETY
B5: ABSENTEEISM

QUESTION B5 - ABSENTEEISM

What is the absenteeism caused by each pathogen, as a proportion of the total absenteeism due to infectious illness, in Belgium considering the 2010-2016 time period, in your opinion?

FULL DEFINITION: Absenteeism is defined as school- and work absenteeism provoked by the pathogen. Please, consider the total burden of disease caused by each pathogen (not per case, but for all cases) relative to the total absenteeism due to infectious illness in Belgium.

LEVELS (i.e. answer categories):

- Low (<0.1%)
- Moderate (0.1-2.5%)
- High (>2.5%)

Because no pathogen-specific registry exists, percentages of these categories are just indicative.

Important note:

- We are interested in the absenteeism due to the total burden of disease caused by each pathogen (not per case, but for all cases) with respect to the total absenteeism due to infectious illness in Belgium (i.e. percentage of the total burden due to infectious diseases). Examples:
    - Pathogens that provoke influenza-like-illness (influenza, parainfluenza, HMPV, adenovirus, ...) are feared by employers during winter months. These pathogens contribute significantly to the total absenteeism due to infectious illness during winter months.
    - Pathogens that have a very low incidence, contribute in general less to the total absenteeism due to infectious illness in Belgium.
    - Pathogens that specifically provoke disease in young children and/or elderly, in general contribute less to the total absenteeism due to infectious illness in Belgium.
- No pathogen-specific registry of absenteeism in Belgium exists, therefore we are interested in your expert opinion.

For the scoring of this criteria, no default answers are given and we totally rely on your expert opinion.
Available 'help data' for absenteeism can be consulted here.

QUESTION B5 - ABSENTEEISM

Please indicate for which pathogen groups you would like to provide your expert opinion.

	I would like to give my opinion for 1 or more pathogen(s) in this pathogen group	I have no opinion for this pathogen group
**Vaccine preventable pathogens (within the national vaccination program)**	○	○
**Endemic/autochtonous pathogens under surveillance**	○	○
**Imported and/or very rare pathogens under surveillance**	○	○
**Limited surveillance and/or congenital surveillance only**	○	○
**Hospital related infections (including invasive mycosis and AMR surveillance)**	○	○

If you indicate 'I would like to give my opinion …', answer option for the pathogens within the indicated group(s) will appear below.

[FOR REASONS OF CLARITY, THE FULL TABLE OF 98 DISEASE IS NOT SHOWN IN THIS PAPER VERSION OF THE SURVEY. THE TABLE LOOKS LIKE THE FOLLOWING (full expert opinion):]

Disease / pathogen	Low	Moderate	High	Do not know
Disease 1	○	○	○	○
Disease 2	○	○	○	○
Disease …	○	○	○	○

Section B - continuation

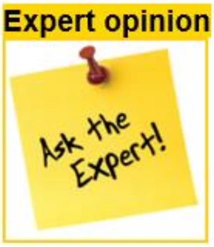

IMPACT ON SOCIETY
B6: HEALTH CARE UTILIZATION

QUESTION B6 – HEALTH CARE UTILIZATION

What is the health-care utilization caused by this pathogen, as a proportion of the total health-care utilization due to infectious illness in Belgium considering the 2010-2016 time period, in your opinion?

FULL DEFINITION: Health-care utilization is defined as the population's use of the health care services (primary care and hospitals) provoked by a pathogen. Health care utilization includes primary care and hospitalization. Please, consider the total burden of disease caused by each pathogen (not per case, but for all cases) relative to the total health care utilization due to infectious illness in Belgium.

NOTE: Infectious disease diagnosis and management comprise a significant part of the workload in primary care. Specifically respiratory tract infections, gastro-intestinal infections and sexually transmitted infections are frequently seen in primary care. In addition, hospitals experience more-and-more patients that suffer from infectious illness (increasing number of patients with immunodeficiency due to cancer, chemotherapy or transplantations, increasing number of elderly, who experience severe infections more frequently than younger people).

LEVELS (i.e. answer categories):

- Low (<0.01%)
- Moderate (0.01-1%)
- High (>1%)

Please note, percentages of these categories are just indicative.

Important note:

- We are interested in the health-care utilization due to the total burden of disease caused by each pathogen (not per case, but for all cases) with respect to the total health-care utilization due to infectious illness in Belgium (i.e. percentage of the total burden due to infectious diseases).
- Health-care utilization cannot be quantified based on a single register, therefore we are interested in your expert opinion.

For the scoring of this criteria, no default answers are given and we totally rely on your expert opinion.
Available 'help data' for health-care utilization can be consulted here.

QUESTION B6 – HEALTH CARE UTILIZATION

Please indicate for which pathogen groups you would like to provide your expert opinion.

	I would like to give my opinion for 1 or more pathogen(s) in this pathogen group	I have no opinion for this pathogen group
**Vaccine preventable pathogens (within the national vaccination program)**	○	○
**Endemic/autochtonous pathogens under surveillance**	○	○
**Imported and/or very rare pathogens under surveillance**	○	○
**Limited surveillance and/or congenital surveillance only**	○	○
**Hospital related infections (including invasive mycosis and AMR surveillance)**	○	○

- **If you indicate 'I would like to give my opinion** …', answer option for the pathogens within the indicated group(s) will appear below.

[FOR REASONS OF CLARITY, THE FULL TABLE OF 98 DISEASE IS NOT SHOWN IN THIS PAPER VERSION OF THE SURVEY. THE TABLE LOOKS LIKE THE FOLLOWING (full expert opinion):]

Disease / pathogen	Low	Moderate	High	Do not know
Disease 1	○	○	○	○
Disease 2	○	○	○	○
Disease …	○	○	○	○

Section B - continuation

IMPACT ON SOCIETY
B7: EXCESS COSTS

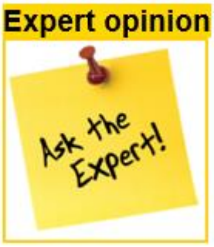

QUESTION B7 – EXCESS COSTS

What proportion of (direct and indirect) excess costs (for patient, society and health care services) is related to infectious illness provoked by this pathogen, in respect to the total excess costs due to infectious illness in Belgium considering the 2010-2016 time period, in your opinion?

FULL DEFINITION: Infectious diseases have considerable economic implications for the society and the individual patients. Excess costs comprise direct and indirect costs for patients and society due to infectious illness. Please, consider the total burden of disease caused by each pathogen (not per case, but for all cases) relative to the total excess costs due to infectious illness in Belgium.

- Costs for the patients include direct costs for health-care and laboratory services and medication. Indirect costs for the patients include transportation and income loss.
- Costs for the society include reimbursements by health-care insurances, other costs covered by health-care insurances, financing of hospital services and laboratory services. Indirect costs for the society include loss of productivity.
- Economic losses related to disease in animals and the loss of food production are NOT included in this question.

LEVELS (i.e. answer categories):

- Low (<0.01%)
- Moderate (0.01-1%)
- High (>1%)

Please note, percentages of these categories are just indicative.

Important note:

- We are interested in the excess cost due to the total burden of disease caused by each pathogen (not per case, but for all cases) with respect to the total excess cost due to infectious illness in Belgium (i.e. percentage of the total burden due to infectious diseases).
- We consider the 2010-2016 period and the prevention strategies/control measures/treatment options/... (e.g. degree of vaccination) that were in place during this time period should be taken into account when answering this question.
- Excess costs due to infectious illness cannot readily be quantified based on available data. Therefore, we are interested in your expert opinion. For example, some pathogens with a considerable incidence are well-known for their high treatment costs (Hepatitis C Virus).

For the scoring of this criteria, no default answers are given and we totally rely on your expert opinion. Available data about excess costs due to infectious illness in Belgium can be consulted here. However, for most pathogens, no data is available. For the others, it is important to mention that quantification methods are not standardized and the factors included in the cost estimates may differ per analysis.

QUESTION B7 – EXCESS COSTS

Please indicate for which pathogen groups you would like to provide your expert opinion.

	**I would like to give my opinion for 1 or more pathogen(s) in this pathogen group**	**I have no opinion for this pathogen group**
**Vaccine preventable pathogens (within the national vaccination program)**	○	○
**Endemic/autochtonous pathogens under surveillance**	○	○
**Imported and/or very rare pathogens under surveillance**	○	○
**Limited surveillance and/or congenital surveillance only**	○	○
**Hospital related infections (including invasive mycosis and AMR surveillance)**	○	○

- **If you indicate 'I would like to give my opinion** …', answer option for the pathogens within the indicated group(s) will appear below.

[FOR REASONS OF CLARITY, THE FULL TABLE OF 98 DISEASE IS NOT SHOWN IN THIS PAPER VERSION OF THE SURVEY. THE TABLE LOOKS LIKE THE FOLLOWING (full expert opinion):]

Disease / pathogen	Low	Moderate	High	Do not know
Disease 1	○	○	○	○
Disease 2	○	○	○	○
Disease …	○	○	○	○

toute une vie en bonne santé

Section B - continuation

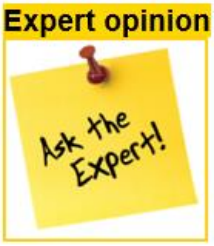

IMPACT ON SOCIETY
B8: PUBLIC ATTENTION

QUESTION B8 – PUBLIC ATTENTION

What is the public attention (media attention, political attention and risk perception among the general public) that is given to each pathogen in Belgium, considering the 2010-2016 time period, in your opinion?

FULL DEFINITION: Risk perception among the general population, amount of media attention and ranking on the political agenda. Infectious diseases are subject of media attention and political attention to various degrees. The public attentions may be provoked by the occurrence of a single case for some pathogens or an exceptional event for other pathogens. Public attention includes a high risk perception for the pathogen among the general public. In addition, it includes pathogens that are high on the political agenda.

<u>LEVELS (i.e. answer categories):</u>

- Low
- Moderate
- High

Important note:

- Please consider the <u>total burden of disease</u> provoked by this pathogen (all cases).
- <u>For the pathogens for which no cases were reported</u>, please, consider the scenario in which a case is occurring in Belgium.

As this variable is not easy to quantify, we rely on your expert opinion to indicate the degree of public attention per pathogen. **No help data is available.**

sciensano

QUESTION B8 – PUBLIC ATTENTION

Please indicate for which pathogen groups you would like to provide your expert opinion.

	I would like to give my opinion for 1 or more pathogen(s) in this pathogen group	I have no opinion for this pathogen group
**Vaccine preventable pathogens (within the national vaccination program)**	○	○
**Endemic/autochtonous pathogens under surveillance**	○	○
**Imported and/or very rare pathogens under surveillance**	○	○
**Limited surveillance and/or congenital surveillance only**	○	○
**Hospital related infections (including invasive mycosis and AMR surveillance)**	○	○

- **If you indicate 'I would like to give my opinion** …', answer option for the pathogens within the indicated group(s) will appear below.

[FOR REASONS OF CLARITY, THE FULL TABLE OF 98 DISEASE IS NOT SHOWN IN THIS PAPER VERSION OF THE SURVEY. THE TABLE LOOKS LIKE THE FOLLOWING (full expert opinion):]

Disease / pathogen	Low	Moderate	High	Do not know
Disease 1	○	○	○	○
Disease 2	○	○	○	○
Disease …	○	○	○	○

Section B - continuation

Default answers

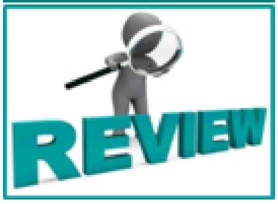

IMPACT ON PUBLIC HEALTH
B9: SPREADING POTENTIAL

QUESTION B9 – SPREADING POTENTIAL:

What is the perceived spreading potential/ contagiousness for each pathogen in Belgium considering the 2010-2016 time period, in your opinion?

FULL DEFINITION: Perceived spreading potential of the pathogen. Indicators for the spreading potential are the theoretical reproductive number of the pathogen (R0: the reproduction of infections in a completely homogeneous and susceptible population), the mode of transmission (transmission by aerosols or droplets usually indicates high spreading potential) and prevention possibilities (e.g. vaccines).

LEVELS (i.e. answer categories):
We have categorized the pathogens according to the R0, however when this information was not available or contradictory, the categorization was based on the mode of transmission and some other contextual information, according to the following operational definitions (i.e. answer categories):

- Low spreading potential
    - R0<1 or;
    - Foodborne pathogens without efficient faecal-oral transmission,
    - Vector borne pathogens when the vector is not present in Belgium or the pathogen is present in low frequencies,
    - Sexual transmitted diseases (STI) with low-moderate transmission efficacies;
    - All other diseases with no human-to-human transmission
- Moderate spreading potential
    - R0>1 or;
    - Foodborne pathogens with efficient faecal-oral transmission,
    - Vector borne pathogens when pathogen or vector is present in high frequencies,
    - Sexual transmitted diseases (STI) with efficient transmission,
    - Direct-contact transmission with low-moderate efficacies
- High spreading potential
    - R0>5 or;
    - Efficient direct-contact transmission,
    - Airborne transmission

Important note:

- The R0 represents the theoretical reproductive number of the infection in a completely susceptible population, that is completely homogeneous.

The following default answers are proposed.
Available 'help data' for the contagiousness and the risk of spread per pathogen can be consulted here.

SPREADING - VACCINE PREVENTABLE PATHOGENS (NATIONAL VACCINATION PROGRAM):

Low	Moderate	High
Clostridium tetani	Corynebacterium diphtheriae, Corynebacterium ulcerans, Corynebacterium pseudotuberculosis	Mumps
	Haemophilus influenza B	Bordetella pertussis (kinkhoest)
	Hepatitis B virus	Measles
	HPV	Poliovirus
	Influenza	Rotavirus
	Invasive pneumo in adults (16+y)	Rubella (congenital)
	Invasive pneumo in children (≤15y)	
	Meningococcus (Neisseria meningitidis)	

SPREADING - ENDEMIC/AUTOCHTHONOUS PATHOGENS:

Low	Moderate	High
Anaplasma	Borrelia burgdorferi senu lato (Lyme disease)	Adenovirus
Bartonella (B. henselae)	Burkholderia cepacia complex	Humaan metapneumovirus (HMPV)
Campylobacter	Cryptosporidium sp.	Human parainfluenzavirus
Chlamydia psittaci (parrot fever)	Entamoeba histolytica	Mycobacterium tuberculosis
Chlamydia trachomatis +LVG	Giardia	Non-polio enteroviruses and parechoviruses
Coxiella burnetii (Q fever)	Hantavirus	Respiratoir syncitieel virus (RSV)
Cyclospora	HAV	
Echinococcus multilocularis	HIV	
HEV	Legionella pneumophila	
Leptospira	Mycoplasma pneumoniae	
Listeria monocytogenes	Noroviruses	
Neisseria gonorrhoeae	Shiga-toxin producing E. coli	
Salmonella (non-typhoid)	Streptococcus pyogenes (SGA/GAS) (invasive)	
Shigella	Hepatitis C virus	
Treponema pallidum		
Yersinia enterocolitica and Yersinia pseudotuberculosis		

SPREADING - IMPORTED AND/OR VERY RARE PATHOGENS:

Low	Moderate	High
Brucella	Coronavirus including MERS, SARS	Bacillus anthracis
Burkholderia mallei/pseudo mallei	Ebola	Smallpox
Chikungunya (arbovirus)	Salmonella Typhoid	
Clostridium botulinum	Vibrio cholerae and Vibrio parahaemolyticus	
Dengue (arbovirus)		
Francisella tularensis (tularaemia)		
Leishmania		
Mycobacterium leprae		
Plasmodium (malaria)		
Rabies virus		
Rickettsia		
Tick-borne encephalitis (arbovirus)		
Trichinosis / trichinellosis		
Tropheryma whipplei		
Trypanosoma cruzi (Chagas disease)		
VCJD		
West Nile virus (arbovirus)		
Yellow fever virus (arbovirus)		
Zika (arbovirus)		

SPREADING - LIMITED SURVEILLANCE AND/OR CONGENITAL SURVEILLANCE ONLY:

Low	Moderate	High
Babesia	Chlamydia pneumoniae	Herpes zoster varicella (zona, varicella) (in CSF)
Bacillus cereus	CMV (congenital)	Parvovirus B19 (congenital)
Pasteurella	Helicobacter pylori	
Toxoplasmose (congenital)	Herpes simplex (in CSF)	
	Scabies	
	Streptococcus agalactiae (GBS) (invasive and/or congenital)	

SPREADING - HOSPITAL RELATED INFECTIONS (INCL. INVASIVE MYCOSIS & AMR):

Low	Moderate	High
Aspergillus (invasive)	Acinetobacter (invasive) (including MDR)	x
Cryptococcus (invasive)	Candida spp (invasive)	
	Clostridium difficile	
	E. coli (non STEC/EHEC) invasive	
	Enterococci (including VRE) (E. faecalis and E. faecium) (invasive)	
	Klebsiella (invasive)	
	Pseudomonas (including MDR)	
	Staphylococcus aureus (including MRSA) (invasive)	

QUESTION B9 – SPREADING POTENTIAL:
Do you agree with the default answers in the tables above (based on available data), or do some pathogens belong to another category in your opinion?

We rely on your expert opinion because:

- It is not possible to base the value of contagiousness on one or few quantitative variables that are available for all pathogens.
- R0 reflects a theoretical situation with ideal circumstances for the pathogen, that does not reflects the real Belgian situation (e.g. when high vaccination rates exist or acquired resistance after exposure exist).
- Conceived contagiousness might differ from our operational definitions above for some pathogens.

Please select the appropriate response for each line:

	I agree on the default answers for all pathogens in this group	I would like to change the answer for 1 or more pathogen(s) in this group	I have no opinion for this group
**Vaccine preventable pathogens (within the national vaccination program)**	○	○	○
**Endemic/autochtonous pathogens under surveillance**	○	○	○
**Imported and/or very rare pathogens under surveillance**	○	○	○
**Limited surveillance and/or congenital surveillance only**	○	○	○
**Hospital related infections (including invasive mycosis and AMR surveillance)**	○	○	○

We would like to know whether:

- you agree with the default answers;
- you like to change the default answers of certain pathogen(s) within certain pathogen group(s);
- you do not have an opinion.

Please, indicate one of these three options for each group of pathogens.

If you indicate that you want to change one or more pathogens within a certain pathogen group, please indicate the corrections in the question(s) that appear upon selection of the pathogen group(s). Again, you have the option for each pathogen to:

- confirm the default answers (leave the default answer unchanged)
- correct the default answer (change the default answer to another category)
- or indicate that you do not know.

[FOR REASONS OF CLARITY, THE FULL TABLE OF 98 DISEASE IS NOT SHOWN IN THIS PAPER VERSION OF THE SURVEY. THE TABLE LOOKS LIKE THE FOLLOWING (pre-filled according to default answer tables above):]

Disease / pathogen	Low	Moderate	High	Do not know
**Disease 1**	○	○	○	○
**Disease 2**	○	○	○	○
**Disease …**	○	○	○	○

Section B - continuation

Expert opinion

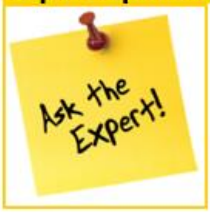

IMPACT ON PUBLIC HEALTH
B10: EVENTS REQUIRING PH ACTION

QUESTION B10 – EVENTS REQUIRING PUBLIC HEALTH ACTION

Which proportion of events provoked by the pathogen requires public health actions in Belgium considering the 2010-2016 time period, in your opinion?

FULL DEFINITION: One of the objectives of the surveillance of infectious diseases is to respond with public health actions upon events. This criteria is defined as the percentage of events provoked by the pathogen that require public health actions.

An event can be defined as: the occurrence of a disease that is unusual with respect to a particular time, place, patient characteristics or circumstances. An event can concern for example:

- A cluster of cases that exceeds significantly the number of cases that is expected for this pathogen in a certain time period and place.
- A cluster of cases that is occurring in an age-group where it is not observed regularly.
- A cluster of cases with a more severe outcome than expected.
- One case may be sufficient to constitute an event for certain infectious diseases (e.g. polio virus).

Public health actions can be defined as: any kind of targeted actions aiming to identify the nature of the event and/or to apply control measures in response to the event (e.g. contact research, investigations to identify a common source of infection).

- For severe and contagious pathogens, every event requires rapid public health actions (e.g. measles, polio virus).
- For less severe pathogens, an event may be noticed and monitored, but direct responses may be deferred and may appear finally unnecessary, when the event was self-limiting and has been resolved without considerable intervention.

LEVELS (i.e. answer categories):

- Small proportion (<25%)
- Moderate proportion (25-75%)
- Large proportion (>75%)

Please note, percentages of these categories are indicative.

For the scoring of this criteria, no default answers are given and we completely rely on your expert opinion.
No help data is available. **No help data is available.**

QUESTION B10 – EVENTS REQUIRING PUBLIC HEALTH ACTION

Please indicate for which pathogen groups you would like to provide your expert opinion.

	I would like to give my opinion for 1 or more pathogen(s) in this pathogen group	I have no opinion for this pathogen group
Vaccine preventable pathogens (within the national vaccination program)	○	○
Endemic/autochtonous pathogens under surveillance	○	○
Imported and/or very rare pathogens under surveillance	○	○
Limited surveillance and/or congenital surveillance only	○	○
Hospital related infections (including invasive mycosis and AMR surveillance)	○	○

- **If you indicate 'I would like to give my opinion** …', answer option for the pathogens within the indicated group(s) will appear below.

[FOR REASONS OF CLARITY, THE FULL TABLE OF 98 DISEASE IS NOT SHOWN IN THIS PAPER VERSION OF THE SURVEY. THE TABLE LOOKS LIKE THE FOLLOWING (full expert opinion):]

Disease / pathogen	Small	Moderate	Large	Do not know
Disease 1	○	○	○	○
Disease 2	○	○	○	○
Disease …	○	○	○	○

Section B - continuation

For Information

IMPACT ON PUBLIC HEALTH
B11: SURVEILLANCE NEEDS

SECTION B11 – SURVEILLANCE NEEDS
FOR INFORMATION – COMMENTS ON TABLES ARE WELCOME

In addition to the variables presented before, we defined various surveillance needs. These needs reflect objectives of possible surveillance systems. The objective of our study is to rank the pathogens based on their impact on public health and importance for surveillance, and therefore the surveillance needs will be included in our analysis. The tables below are provided just for your information. We will not explicitly ask you to review the tables, because most values are rather objective and already reviewed by internal experts. However if you do not agree with this information you can indicate your concerns in the comment box below.

A detailed definition of each surveillance need can be consulted here.

According to the pathogen characteristics, the surveillance needs per pathogen in the Belgian context are as follows:

SURVEILLANCE NEEDS - VACCINE PREVENTABLE PATHOGENS (NATIONAL VACCINATION PROGRAM):

Pathogen	International obligations for surveillance	WHO objective for eradication or elimination	Vaccine included in NVP	Risk for vaccine-triggered strain replacement	Existing multi-resistance against anti-infective drugs	Congenital risks	NRC/NRL essential for the diagnosis
Mumps	+		+				
Bordetella pertussis	+		+		+		
Clostridium tetani	+		+				
Corynebacterium diphtheriae, Corynebacterium ulcerans, Corynebacterium pseudotuberculosis	+		+		+		+
Haemophilus influenza B	+		+		+		
Hepatitis B virus	+	+	+		+	+	
HPV			+	+			
Influenza	+		+		+		
Invasive pneumo in adults (16+)	+		+	+	+		
Invasive pneumo in children (≤15 years)	+		+	+	+		
Measles	+	+	+			+	+
Meningococcus (Neisseria meningitidis)	+		+				
Poliovirus	+	+	+				+
Rotavirus	+		+	+			
Rubella (congenital)	+	+	+			+	+

SURVEILLANCE NEEDS - ENDEMIC/AUTOCHTHONOUS PATHOGENS:

Pathogen	International obligations for surveillance	WHO objective for eradication or elimination	Vaccine included in NVP	Risk for vaccine-triggered strain replacement	Existing multi-resistance against anti-infective drugs	Congenital risks	NRC/NRL essential for the diagnosis
Adenovirus						+	
Anaplasma							+
Bartonella (B. henselae)							+
Borrelia burgdorferi senu lato	+						+
Burkholderia cepacia complex					+		
Campylobacter	+				+		
Chlamydia psittaci (parrot	+				+		
Chlamydia trachomatis +LVG	+						
Coxiella burnetii (Q fever)	+						
Cryptosporidium sp.	+						
Cyclospora							+
Echinococcus multilocularis	+						+
Entamoeba histolytica	+						+
Giardia	+						
Hantavirus	+						
HAV	+						
Hepatitis C virus	+	+			+	+	
HEV							+
HIV	+				+	+	
Human metapneumovirus (HMPV)							
Human parainfluenzavirus							
Legionella pneumophila	+				+		+
Leptospira	+						+
Listeria monocytogenes	+					+	
Mycobacterium tuberculosis	+				+		+
Mycoplasma pneumoniae					+		
Neisseria gonorrhoeae	+				+		
Non-polio enteroviruses and parechoviruses							
Noroviruses							
Respiratoir syncitieel virus (RSV)							
Salmonella (non-typhoid)	+				+		
Shiga-toxin producing E. coli	+				+		+
Shigella	+				+		
Streptococcus pyogenes					+		
Treponema pallidum	+					+	
Yersinia enterocolitica, pseudotuberculosis	+				+		

SURVEILLANCE NEEDS - IMPORTED AND/OR VERY RARE PATHOGENS:

Pathogen	International obligations for surveillance	WHO objective for eradication or elimination	Vaccine included in NVP	Risk for vaccine-triggered strain replacement	Existing multi-resistance against anti-infective drugs	Congenital risks	NRC/NRL essential for the diagnosis
Bacillus anthracis	+						+
Brucella.spp	+						+
Burkholderia mallei/pseudo mallei	+						+
Chikungunya (arbovirus)	+						+
Clostridium botulinum	+				+		+
Coronavirus including MERS, SARS	+						+
Dengue (arbovirus)	+						+
Ebola	+						+
Francisella tularensis (tularaemia)	+						+
Leishmania	+				+		+
Mycobacterium leprae	+						+
Plasmodium (malaria)	+				+		
Rabies virus	+						+
Rickettsia							+
Salmonella Typhoid	+				+		+
Smallpox	+						+
Tick-borne encephalitis (arbovirus)	+						+
Trichinosis / trichinellosis	+						+
Tropheryma whipplei							+
Trypanosoma cruzi (Chagas disease)					+		+
VCJD	+						+
Vibrio cholerae and Vibrio parahaemolyticus	+						+
West Nile virus (arbovirus)	+					+	+
Yellow fever virus (arbovirus)	+						+
Zika (arbovirus)	+					+	+

SURVEILLANCE NEEDS - LIMITED SURVEILLANCE AND/OR CONGENITAL SURVEILLANCE ONLY:

Pathogen	International obligations for surveillance	WHO objective for eradication or elimination	Vaccine included in NVP	Risk for vaccine-triggered strain replacement	Existing multi-resistance against anti-infective drugs	Congenital risks	NRC/NRL essential for the diagnosis
Babesia							+
Bacillus cereus							
Chlamydia pneumoniae					+		
CMV (congenital)						+	+
Helicobacter pylori	+						
Herpes simplex (in CSF)						+	
Herpes zoster varicella (zona, varicella) (in CSF)	+					+	
Parvovirus B19 (congenital)						+	
Pasteurella							
Scabies							
Streptococcus agalactiae (GBS) (invasive and/or congenital)					+		
Toxoplasmose (congenital)	+					+	+

SURVEILLANCE NEEDS - HOSPITAL RELATED INFECTIONS (INCL. INVASIVE MYCOSIS AND AMR):

Pathogen	International obligations for surveillance	WHO objective for eradication or elimination	Vaccine included in NVP	Risk for vaccine-triggered strain replacement	Existing multi-resistance against anti-infective drugs	Congenital risks	NRC/NRL essential for the diagnosis
Acinetobacter (invasive) (including MDR)					+		
Aspergillus (invasive)							
Candida spp (invasive)					+		
Clostridium difficile					+		
Cryptococcus (invasive)					+		+
E. coli (non STEC/EHEC) invasive					+		
Enterococci (including VRE) (E. faecalis and E. faecium) (invasive)					+		+
Klebsiella (invasive)					+		
Pseudomonas (including MDR)					+		
Staphylococcus aureus (including MRSA) (invasive)					+		

If you <u>do not</u> agree with the information listed in the tables above, you can indicate this as a suggestion below.

Please write your answer here:

Section C - Future

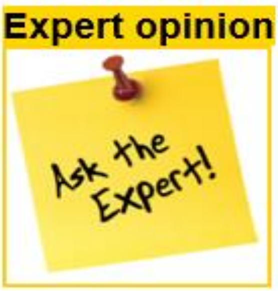

FUTURE RISK
C1: PROBABILITY OF INCREASED IMPACT IN NEXT 10 YEARS

QUESTION C1 – FUTURE RISK

What is the estimated probability that the impact of a pathogen increases in the next 10 years in Belgium, in your opinion?

FULL DEFINITION:
Changes to the pathogen or the environment may lead to a worse threat than it is at present. Such worsening can occur through various mechanisms, for example:

- The evolution of new pathogen traits (e.g. virulence, enhanced transmissibility in humans, antimicrobial resistance)
- Changing vector habitats (i.e. due to climate change)
- Changes in animal reservoirs
- Evolution in human activities (e.g. global trade, travel, etc.)
- Evolution human susceptibility (e.g. ageing, herd immunity, vaccine coverage, etc.)
- Changes in public health capacity

This question includes the probability of introduction of new pathogens into Belgium and the potential for onwards transmission in humans in the next 10 years.

LEVELS (i.e. answer categories):

- Very low (<1%) probability of increased impact
- Low (1-10%) probability of increased impact
- Medium (10-99%) probability of increased impact
- High (>99%) probability of increased impact

Please note, percentages of these categories are indicative.

This question will not be included in the MCDA analysis for the final ranking of the pathogens, but it will allow a broader interpretation of the results. **No help data is available.**

QUESTION C1 – FUTURE RISK

[FOR REASONS OF CLARITY, THE FULL TABLE OF 98 DISEASE IS NOT SHOWN IN THIS PAPER VERSION OF THE SURVEY. THE TABLE LOOKS LIKE THE FOLLOWING (full expert opinion):]

Disease / pathogen	Very Low	Low	Medium	High
**Disease 1**	○	○	○	○
**Disease 2**	○	○	○	○
**Disease …**	○	○	○	○

THANK YOU!

Thank you gratefully for your kind participation! Please, do not forget to SUBMIT!

If you would like to get informed personally about the final results, feel free to leave your email address below:

Email: ____

If you have any comments/feedback/suggestions related to this survey or the ranking of infectious diseases in general, please feel free to write them down below:

CRITERIA DEFINITIONS (FYI)

Surveillance needs:

WHO objective for eradication or elimination: Some diseases are target of specific eradication or elimination programs by the WHO. These disease control programs require targeted surveillance and rapid response capabilities at the national level.

International obligations for surveillance: All diseases that are included in the WHO, ECDC and/or OIE surveillance programs. National surveillance data is reported to the international surveillance programs.

Existing multidrug resistance: The presence of drugs resistance (antibiotics, antivirals, ect) other than those inherent to the specific species. The definition of multidrugs resistance varies per pathogen (e.g. resistance to 3 different drug classes).

Vaccine included in NVP: All pathogens that are included in the national vaccination program (NVP) for the general population. This accounts for the lower incidence of vaccine-preventable diseases due to effective prevention programs and the need to maintain adequate surveillance capacities for these pathogens.

Risk for vaccine triggered strain replacement: Risk for increased incidence of formerly subdominant types or species after vaccination (vaccination can drive the emergence of formerly subdominant strains).

Congenital risk: Risk for mother-to-child transmission of an infectious disease AND serious complications for the child.

NRC/RefLab essential for diagnosis: All pathogens for which a national laboratory is essential for the first diagnosis of the patients AND the patients' treatment. This includes pathogens for which the national laboratory is essential for the diagnosis AND treatment of only a proportion of patients (e.g. difficult cases). This concerns the diagnosis at genus-level and does not include typing or antibiograms of pathogens.

Impact on society:

Work and school absenteeism*: Absenteeism due to the total burden of disease caused by each pathogen (not per case, but for all cases) relative to the total absenteeism due to infectious illness in Belgium.

Excess costs*: Direct and indirect costs due to the total burden of disease caused by each pathogen (not per case, but for all cases) relative to the total excess costs due to infectious illness in Belgium.

Health care utilization*: Health care utilization (primary care and hospitalization) due to the total burden of disease caused by each pathogen (not per case, but for all cases) relative to the total health care utilization due to infectious illness in Belgium.

Public attention*: Risk perception among the general population, amount of media attention and ranking on the political agenda. For infections that did not occur during the reference period, this criteria can be considered as the public attention that the pathogen will attract in the scenario that one case will occur.

**Assessed is the total burden of one infectious disease (all cases, 1 disease) relative to the total burden of all infectious diseases in Belgium (all cases, all infectious diseases).*

Impact on public health:

Spreading potential: Perceived spreading potential of the pathogen. Indicators for the spreading potential are the theoretical reproductive number of the pathogen (R0: the reproduction of infections in a completely homogeneous and susceptible population), the mode of transmission (transmission by aerosols or droplets usually indicates high spreading potential) and prevention possibilities.

Proportion of events requiring public health action: Percentage of events provoked by the pathogen that require urgent public health actions. Event is defined as the occurrence of disease that is unusual and/or exceeding base-line levels with respect to a particular time, place and circumstances. Public health actions are any kind of targeted actions aimed to identify the nature of the event and/or to apply control measures in response to the event.

Impact on the patient:

Case-fatality ratio*: Percentage of lethal cases among all symptomatic cases annually in Belgium.

Severity*: Perceived severity of the pathogen in Belgium, i.e. distribution of the clinical presentation of all symptomatic cases. This represents the discomfort at individual level for the patient.

Chronicity and/or chronic sequelae*: Percentage of patients that experience chronic disease (>6 months) and/or have serious sequelae relative to all symptomatic cases in Belgium.

**Assessed for each particular pathogen in question, relative to all symptomatic cases provoked by that particular pathogen.*

Incidence and trend:

Incidence: Total number of symptomatic cases annually in Belgium. This is not always equal to the number of reported cases by the national surveillance systems. Correction factors for the estimated underdiagnosis and underreporting should be applied, in order to obtain the estimated total number of symptomatic cases. This includes both imported and authochtoneous cases.

Trend: Trend of the incidence for each pathogen in Belgium over the period 2010-2016. This again concerns the trend of the estimated total number of symptomatic cases. This is not always equal to the observed trend in surveillance data (e.g. in case of increased testing for this pathogen over the years).
